# m^6^A regulator-based methylation modification patterns characterized by distinct tumor microenvironment immune profiles in colon cancer

**DOI:** 10.7150/thno.52717

**Published:** 2021-01-01

**Authors:** Wei Chong, Liang Shang, Jin Liu, Zhen Fang, Fengying Du, Hao Wu, Yang Liu, Zhe Wang, Yang Chen, Shengtao Jia, Liming Chen, Leping Li, Hao Chen

**Affiliations:** 1 Department of Gastrointestinal Surgery, Shandong Provincial Hospital Affiliated to Shandong First Medical University, Jinan, Shandong, 250021, China; 2 Department of Gastrointestinal Surgery, Shandong Provincial Hospital, Cheeloo College of Medicine, Shandong University, Jinan, Shandong, 250021, China; 3 Key Laboratory of Engineering of Shandong Province, Shandong Provincial Hospital, Jinan, Shandong, 250021, China; 4 Department of Gastroenterology, Key Laboratory of Engineering of Shandong Province, Shandong Provincial Hospital, Jinan, Shandong, 250021, China; 5 Tianjin Sino-US Diagnostics Co., Ltd, Tianjin, China; 6 Department of Radiation Oncology, Tianjin Medical University Cancer Institute and Hospital, National Clinical Research center for Cancer; Key Laboratory of Cancer Prevention and Therapy; Tianjin's Clinical Research Center for Cancer, Tianjin, China; 7 Department of Tumor Cell Biology, National Clinical Research Center for Cancer, Tianjin's Clinical Research Center for Cancer, Tianjin Medical University Cancer Institute and Hospital, Tianjin, China; 8 Division of Gastroenterology, Department of Medicine, Center for Advanced Biomedical Imaging and Photonics, Beth Israel Deaconess Medical Center, Harvard University, Boston, 02215, MA, USA; 9 Clinical Research Center of Shandong University, Clinical Epidemiology Unit, Qilu Hospital of Shandong University, Jinan, Shandong, 250021, China

**Keywords:** Colon cancer, m^6^A modification, Tumor microenvironment, Immune profiles, Immunotherapy

## Abstract

Recent studies have highlighted the biological significance of RNA N^6^-methyladenosine (m^6^A) modification in tumorigenicity and progression. However, it remains unclear whether m^6^A modifications also have potential roles in immune regulation and tumor microenvironment (TME) formation.

**Methods**: In this study, we curated 23 m^6^A regulators and performed consensus molecular subtyping with NMF algorithm to determine m^6^A modification patterns and the m^6^A-related gene signature in colon cancer (CC). The ssGSEA and CIBERSORT algorithms were employed to quantify the relative infiltration levels of various immune cell subsets. An PCA algorithm based m^6^Sig scoring scheme was used to evaluate the m^6^A modification patterns of individual tumors with an immune response.

**Results**: Three distinct m6A modification patterns were identified among 1307 CC samples, which were also associated with different clinical outcomes and biological pathways. The TME characterization revealed that the identified m^6^A patterns were highly consistent with three known immune profiles: immune-inflamed, immune-excluded, and immune-desert, respectively. Based on the m^6^Sig score, which was extracted from the m^6^A-related signature genes, CC patients can be divided into high and low score subgroups. Patients with lower m^6^Sig score was characterized by prolonged survival time and enhanced immune infiltration. Further analysis indicated that lower m^6^Sig score also correlated with greater tumor mutation loads, PD-L1 expression, and higher mutation rates in SMGs (e.g., *PIK3CA* and *SMAD4*). In addition, patients with lower m^6^Sig scores showed a better immune responses and durable clinical benefits in three independent immunotherapy cohorts.

**Conclusions**: This study highlights that m^6^A modification is significantly associated with TME diversity and complexity. Quantitatively evaluating the m^6^A modification patterns of individual tumors will strengthen our understanding of TME characteristics and promote more effective immunotherapy strategies.

## Introduction

Methylation of N6 adenosine (m^6^A), which is widely observed in mRNAs, lncRNAs and miRNAs, is the most common type of RNA modification and plays crucial roles in multiple physiological processes and disease progression 1, 2. m^6^A modification is also a kind of dynamic and reversible process, which is controlled by different types of regulatory proteins: the methyltransferases (“writers”), the demethylases (“erasers”) and binding proteins (“readers”) 3. The expression and function of these regulatory proteins have great impacts on m^6^A modification, and investigation of these regulators can help understanding the mechanisms of m^6^A in gene regulation 4, 5. Increasing evidence has demonstrated that dysregulated expression and genetic changes of m^6^A regulators are correlated with malignant tumor progression and immunomodulatory abnormalities 6-9. A comprehensive understanding of the genetic variation and expression perturbations underlying cancer heterogeneity will further benefit the identification of RNA methylation-based therapeutic targets 10.

Colon cancer (CC) is one of the most common malignancies and remains the primary cause of cancer death worldwide, and 30% - 50% of patients develop recurrence, metastasis and even death within 5 years of treatment 11, 12. Recently, with the increased understanding of the diversity and complexity of the tumor microenvironment contexture (including cancer cells, stromal cells, infiltrating immune cells, and secreted cytokines *et al.*), the crucial immune cell subsets in tumorigenesis and metastasis have been gradual recognized 13-16. Indeed, assessment of the densities of lymphocyte populations (CD3 and cytotoxic CD8 T cells) at the tumor center and the tumor margin were demonstrated to play an important complementary role to the tumor staging system in relapse and mortality prediction in CC 17. Moreover, current immunotherapies represented by specific immune checkpoint inhibitors (ICIs), such as anti-CTLA-4 and anti-PD-1/L1, have achieved a marked durable response in CC treatment 18, 19. Evaluating immune infiltration based on the characteristics of the TME constitutes a critical approach to predicting the response to existing ICIs and developing novel immunotherapeutic strategies 20-22. Current studies also proposed the novel concept of 'immune contexture', which classified CC tumors into three major immune coordination profiles (hot, excluded and cold) and represented different TME characteristics and treatment options 23-25. Therefore, tumor immune phenotypes identified by comprehensively parsing the components of the tumor microenvironment will assist in guiding and predicting immunotherapeutic responsiveness 17, 26, 27.

Recent studies revealed the interactions between TME immune cell infiltration and m^6^A modification, which cannot be fully explained by the RNA degradation mechanism. Han *et al.* reported that *YTHDF1* promotes lysosomal protease-directed degradation of tumor neoantigens in dendritic cells by recognizing their m^6^A modification and enhancing their translation 28. Loss of *YTHDF1* in dendritic cells markedly enhances the cross-presentation of antigens and the cross-priming of CD8^+^ T cells. Another study demonstrated that *FTO* impeded interferon-gamma (IFN-γ)-induced cytotoxicity in melanoma cells in vitro by upregulating *PD-1, CXCR4,* and *SOX10* through suppression of *YTHDF2*-mediated RNA decay process. Moreover, knockdown of* FTO* sensitized melanoma to anti-PD-1 treatment in mice via the upregulation of IFN-γ 29. *METTL3*-mediated mRNA m^6^A modification is essential for translation of the costimulatory molecules *CD40, CD80*, and the Snail homeostasis in cancer progression 30, 31. However, the aforementioned studies were all restricted to one or two m^6^A regulators on account of technical limitations, while the antitumor effect of these regulators is regulated by numerous tumor suppressor factors that act in a highly coordinated manner. As an alternative, continuously accumulating transcriptomics and genomic data provide an ideal resource for comprehensive analysis of m^6^A regulators and immune regulation 32, 33. Thus, recognizing the TME cell infiltration characteristics mediated by multiple m^6^A regulators will contribute to enhancing our understanding of cancer immunity.

In this study, we comprehensively evaluated the association between m^6^A modification patterns and TME cell-infiltrating characteristics by integrating the transcriptomic and genomic data of 1307 CC samples from TCGA and GEO databases. Three distinct m^6^A modification patterns with nonnegative matrix factorization (NMF) clustering were identified, and the TME characteristics of these three patterns were closely linked to three previously reported immune phenotypes: immune-inflamed, immune-excluded, and immune-desert 25. Moreover, we constructed a scoring scheme to quantify the m^6^A modification patterns of individual tumors and predict patients' clinical response to ICI treatment. These findings suggested that m^6^A modification plays an indispensable role in shaping diverse tumor immune microenvironment profiles and in directing therapeutic intervention plans for colon cancer.

## Methods

### Collect and preprocess of publicly attainable expression datasets

Gene expression data and clinical features of CC samples were retrospectively collected from publicly available datasets of the NCBI GEO database (https://www.ncbi.nlm.nih.gov/geo/) and TCGA (https://cancergenome.nih.gov/). The selection criteria of CC datasets were adopted from the workflow of Dai *et al.* 34, and a total of 1307 patients were enrolled for analysis, including those from the GSE39582/CIT Cohort (N = 557) 35, GSE37892 (N = 130) 36, GSE14333 (N = 226) 37, and TCGA-COAD (The Cancer Genome Atlas-Colon Adenocarcinoma, N = 394) datasets (Table S1). The GSE17538 (N = 200) 38 dataset was excluded from this analysis owing to its probe cell intensity (CEL) files extensively overlapped with the GSE14333 series. Since these GEO datasets shared the same microarray sequencing platform (Affymetrix HG-U133 plus 2.0), we downloaded the raw “CEL” files and performed background adjustment and quantile normalization by 'affy' and 'simpleaffy' packages. TCGA RNA sequencing data (FPKM format) were downloaded from the UCSC Xena (https://gdc.xenahubs.net/download/TCGA-COAD.htseq_fpkm.tsv.gz) and transformed into transcripts per kilobase million (TPM) format. The ComBat method from the 'SVA' R package was used to remove the batch effects among different GEO datasets 34. The genomic mutation data (including somatic mutation and copy number variation) of TCGA-COAD were curated from the UCSC Xena database and the Davoli *et al.* study 39. The R package 'Rcircos' was employed to plot the copy number variation landscape of 23 m^6^A regulators in human chromosomes. Nonsynonymous mutation (including frameshift mutation, inflame mutation, missense mutation, nonsense mutation, and splice site mutation) counts were recognized as tumor mutation load (TML). The clinical information and m^6^A regulator expression of the meta-GEO and TCGA datasets are listed in Table S2 and S3.

### Consensus molecular clustering of twenty-three m^6^A regulators by NMF

We retrieved the literatures related to m^6^A methylation modification, and a total of 23 acknowledged m^6^A regulator genes were curated and analyzed to identify distinct m^6^A methylation modification patterns 1-3, 6. The 23 m^6^A regulators included 8 writers (*CBLL1, KIAA1429, METTL14, METTL3, RBM15, RBM15B, WTAP,* and *ZC3H13*), 2 erasers (*ALKBH5* and *FTO*), and 13 readers (*ELAVL1, FMR1, HNRNPA2B1, HNRNPC, IGF2BP1, IGF2BP2, IGF2BP3, LRPPRC, YTHDC1, YTHDC2, YTHDF1, YTHDF2,* and *YTHDF3*). We performed consensus clustering with NMF to identify distinct m^6^A modification patterns based on the expression of 23 m^6^A regulators. Specifically, the expression of 23 m^6^A regulators (Matrix A) was factorized into 2 nonnegative matrices W and H (i.e., A≈WH). Repeated factorization of matrix A was performed, and its outputs were aggregated to obtain consensus clustering of colon cancer samples. The optimal number of clusters was selected according to cophenetic, dispersion, and silhouette coefficients. The R package 'NMF' (version 0.22.0) with the brunet algorithm and 200 nruns was used to perform the consensus clustering.

### Gene set variation analysis (GSVA) and Gene Ontology (GO) annotation

We utilized GSVA analysis 40 with the R package 'GSVA' to investigate the variation in biological processes between different m^6^A modification patterns. The well-defined biological signatures were derived from the Hallmarker gene set 41 (download from MSigDB database v7.1) and Mariathasan *et al.* constructed gene set 27 (curated from IMvigor210CoreBiologies packages). GO annotation for m^6^A phenotype-related genes was performed in the R package 'clusterProfiler' with the cutoff value of FDR < 0.01.

### Immune cell infiltration estimation by ssGSEA and deconvolution algorithm

Single sample gene set enrichment analysis (ssGSEA) was introduced to quantify the relative abundance of 28 immune cell types in the tumor microenvironment. Special feature gene panels for marking each immune cell type were curated from a recent study 42, 43. The relative abundance of each immune cell type was represented by an enrichment score in ssGSEA analysis and normalized to unity distribution from 0 to 1. The biosimilarity of the infiltrating immune cells was estimated by multidimensional scaling (MDS) and a Gaussian fitting model. The deconvolution approach CIBERSORT 44 (http://cibersort.stanford.edu/) was used to estimate the abundances of 22 distinct leukocyte subsets with the gene expression profile of colon cancer.

### Quantify the immune response predictor: Immunophenoscore, TIDE and ESTIMATE

Immunophenoscore (IPS) is a superior predictor of response to anti-CTLA-4 and anti-PD-1 regimens, which quantify the determinants of tumor immunogenicity and characterize the intratumoral immune landscapes and cancer antigenomes 42. The scoring scheme developed from a panel of immune-related genes belonging to the four classes: MHC-related molecules (MHC), checkpoints or immunomodulators (CP), effector cells (EC) and suppressor cells (SC). The weighted averaged Z score was calculated by averaging the samplewise Z scores of the four classes within the respective category and the sum of the weighted averaged Z score was calculated as the IPS. The Tumor Immune Dysfunction and Exclusion (TIDE) algorithm proposed by Jiang *et al.* was utilized to model distinct tumor immune evasion mechanisms 45, including dysfunction of tumor infiltration cytotoxic T lymphocytes (CTLs) and exclusion of CTLs by immunosuppressive factors. A higher TIDE score indicated tumor cells more likely to induce immune escape, thus indicating a lower response rate to ICI treatment. The Estimation of Stromal and Immune Cells in Malignant Tumors using Expression Data (ESTIMATE) algorithm 46, which takes advantage of the unique properties of the transcriptional profiles to infer the tumor cellularity as well as the tumor purity. By using the ESTIMATE algorithm, we calculated the immune and stromal scores to predict the level of infiltrating immune and stromal cells and these form the basis to infer tumor purity. Tumor tissues with abundant immune cell infiltration represented a higher immune score and lower level of tumor purity.

### Significantly mutated genes and tumor mutational signatures

We utilized the MutSigCV algorithm to identify significantly mutated genes (SMGs) 47, 48. Specifically, MutSigCV measures the significant enrichment of nonsilent somatic mutations in a gene by addressing mutational context-specific background mutation rates. Genes with statistically significant (q < 0.1) and certified in the human Cancer Cell lines Encyclopedia (CCLE) 49 were recognized as SMGs 50 (Table S4). The mutational landscape of m^6^A modification genes and SMGs in TCGA-COAD cohort was depicted by the waterfall function of the R 'maftools' package 51. Mutational signatures extracted from the TCGA genomic data also adopted the 'maftools' package. ExtractSignatures function based on Bayesian variant nonnegative matrix factorization, factorized the mutation portrait matrix into two nonnegative matrices '**signatures'** and** 'contributions'**, where '**signatures'** represent mutational processes and **'contributions'** represent the corresponding mutational activities 52. The SignatureEnrichment function can automatically determine the optimal number of extracted mutational signatures and assign them to each sample based on the mutational activities. The extracted mutational portrait of CC was compared and annotated by cosine similarity analysis against the Catalogue of Somatic Mutations in Cancer (COSMIC) 53.

### Identification of DEGs between distinct m^6^A modification phenotypes

The previous consensus clustering algorithm classified patients into three distinct m^6^A modification patterns, and we next determined m^6^A modification-related differentially expressed genes (DEGs) among distinct m^6^A phenotypes. The R package 'limma' 54 was used to evaluate DEGs in CC samples between different modification clusters. Specifically, gene expression data were normalized by voom and then fed to lmFit and eBayes functions to calculate the differential expressed statistics. The significance filtering criteria of DEGs were set as an adjusted P value less than 0.001.

### Construction of the m^6^Sig score

We developed an m^6^A scoring scheme to quantify the m^6^A modification level of individual patients by using principal component analysis (PCA). Specifically, the overlapping DEGs identified from different m^6^A clusters were selected and employed to perform prognostic analysis for each gene using a univariate Cox regression model. The genes with a significant prognostic impact were extracted for further feature selection by using recursive feature elimination (RFE) with random forest and the 10-fold cross-validation method in the 'caret' package. We then curated the expression profile of the final determined genes to perform PCA analysis, and principal components 1 and 2 were extracted and served as the signature score. This method mainly focuses on the score on the set with the largest block of well correlated (or inverse-correlated) genes in the set, while downweighting contributions from genes that do not track with other set members. We then adopted a formula similar to previous studies to define the m^6^Sig score 55, 56: m^6^Sig score= ∑(PC1_i_+PC2_i_), where is the expression of final determined m^6^A phenotype-related genes.

### Collection of genomic and clinical information of the ICI-based cohort

We systematically searched the gene expression profiles of ICI therapy, which could be publicly obtained and coupled with detailed clinical pathology information. Three immunotherapeutic cohorts were finally included in our study: metastatic melanoma treated with nivolumab (anti-PD-1 mcAb) 57 or ipilimumab (anti-CTLA-4 mcAb) 58, and metastatic urothelial cancer (mUC) treated with atezolizumab (anti-PD-L1 mcAb) 27. The gene expression profiles of pre-therapy biopsy samples were curated and transformed into the TPM format for further analysis.

### Statistical analyses

The statistical analyses in this study were generated by R-3.6.1. For quantitative data, statistical significance for normally distributed variables was estimated by Student's t-tests, and nonnormally distributed variables were analyzed by the Wilcoxon rank-sum test. For comparisons of more than two groups, Kruskal-Wallis tests and one-way analysis of variance were used as nonparametric and parametric methods, respectively 59. Two-sided Fisher exact tests were used to analyze contingency tables. Kaplan-Meier survival analysis and the Cox proportional hazards model were used to analyze the association between the m^6^A modification pattern and prognosis with the R package 'Survminer' (0.4.6). The surv-cutpoint function from the 'survival' package was applied to stratify samples into high and low m^6^Sig score subgroups. The receiver operating characteristic (ROC) curve was used to assess the prognosis classification performance of the m^6^Sig score model, and the area under the curve (AUC) were calculated using 'timeROC' package (0.3). Patients with detailed clinical information were included and adjusted for confounding factors in the multivariate regression model. All comparisons were two-sided with an alpha level of 0.05, and the Benjamini-Hochberg method was applied to control the false discovery rate (FDR) for multiple hypothesis testing 60.

## Results

### Landscape of genetic variation of m^6^A regulators in colon cancer

**Figure 1:**
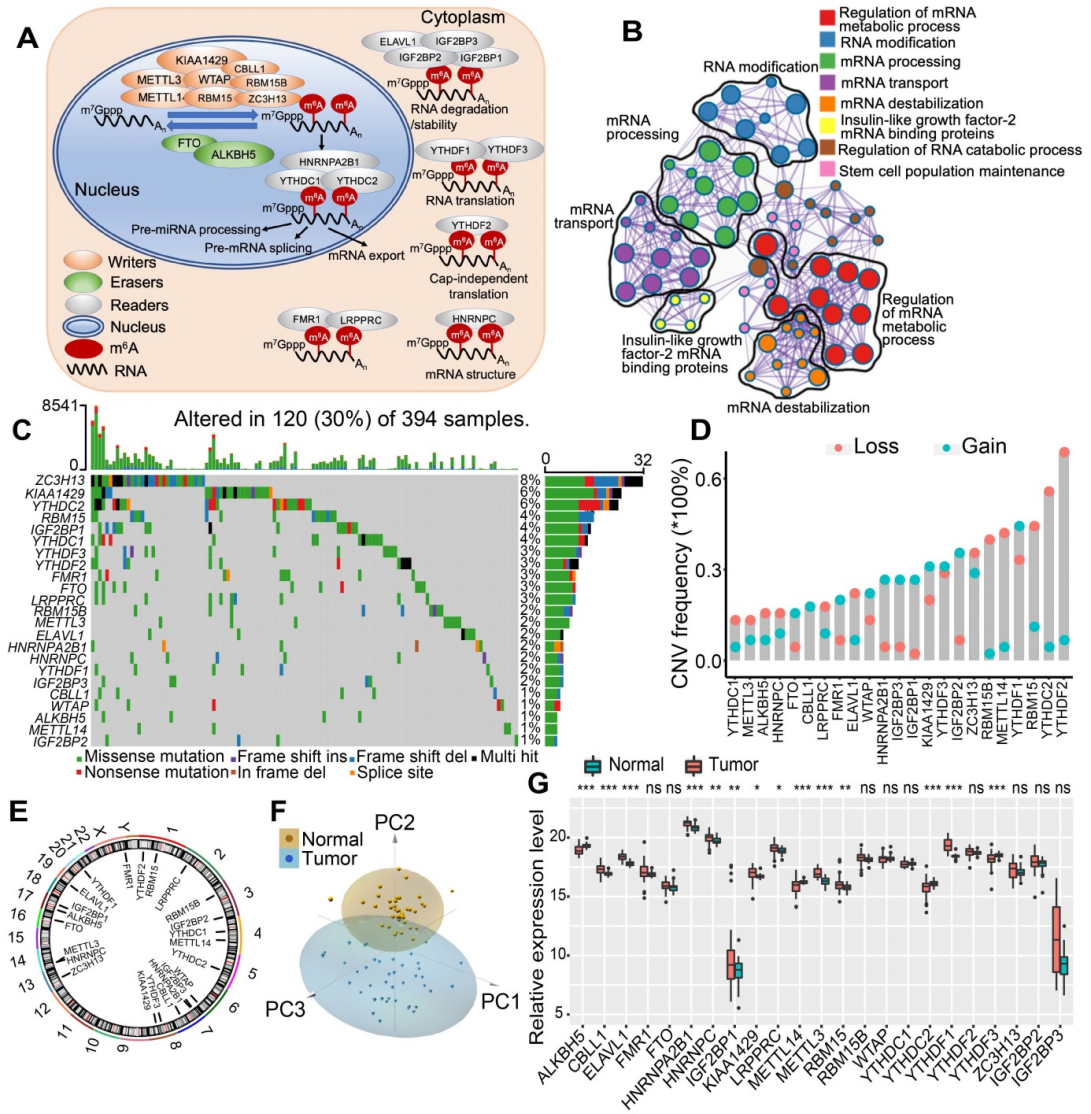
** The landscape of genetic alterations of m^6^A regulators in colon cancer.** (A) Regulation of m^6^A modification and its biological functions in RNA metabolism by m^6^A “writer”, “eraser” and “reader” proteins. m^6^A RNA methylation was known to be involved in all stages in the life cycle of RNA including pre-mRNA splicing, pre-miRNA processing, RNA translation, RNA degradation/stability, etc. (B) Metascape enrichment network visualization showed the intra-cluster and inter-cluster similarities of enriched terms, up to 20 terms per cluster. Cluster annotations were shown in the color code. (C) 120 of the 394 CC patients experienced genetic alterations of 23 m^6^A regulators, with a frequency of 30%, mostly including amplification, missense mutations, and deep deletions. The number on the right indicated the mutation frequency in each regulator. Each column represented individual patients. (D) The CNV mutation frequency of 23 m^6^A regulators was prevalent. The column represented the alteration frequency. The deletion frequency, pink dot; The amplification frequency, blue dot. (E) The location of CNV alteration of m^6^A regulators on chromosomes. (F) Principal component analysis of 23 m^6^A regulators to distinguish tumors from normal samples. (G) The difference of mRNA expression levels of 23 m^6^A regulators between normal and CC samples. The asterisks represented the statistical P-value (*P < 0.05; **P < 0.01; ***P < 0.001).

In this study, we investigated the roles of 23 m^6^A RNA methylation regulatory genes in CC (“writers”: CBLL1, KIAA1429, METTL14, METTL3, RBM15, RBM15B, WTAP, and ZC3H13; “readers”: ELAVL1, FMR1, HNRNPA2B1, HNRNPC, IGF2BP1, IGF2BP2, IGF2BP3, LRPPRC, YTHDC1, YTHDC2, YTHDF1, YTHDF2, and YTHDF3; and “erasers”: ALKBH5 and FTO). Figure 1A shows that the dynamic reversible process of these m^6^A regulators can recognize, remove and add m^6^A-modified sites and alter substantial biological processes, such as RNA splicing, RNA translation, and RNA degradation. GO enrichment and Metascape analyses of 23 m^6^A regulators were conducted, and significantly enriched biological processes are summarized in Figure 1B and Figure S1A. We first determined the prevalence of somatic mutations of 23 m^6^A regulators in CC. A total of 120 of the 394 (30.5%) samples experienced genetic alterations of m^6^A regulators, primarily including amplification, missense mutations, and deep deletions. ZC3H13 showed the highest mutation frequency, followed by KIAA1429 and YTHDC2 (Figure 1C). We also examined mutation co-occurrence across all m^6^A regulators and found a significant mutation co-occurrence relationship between IGF2BP1 and YTHDC2, YTHDF1 and KIAA1429, LRPPRC and YTHDF2, and RBM15 and ZC3H13 (Figure S1B). Further analysis of 23 m^6^A regulators revealed that CNV mutations were prevalent. YTHDF1/3, IGF2BP1/2/3, KIAA1429 and HNRNPA2B1 showed widespread CNV amplification. In contrast, YTHDF2, YTHDC2, METTL14, RBM15 and ZC3H13 had prevalent CNV deletions (Figure 1D). The locations of CNV alterations of 23 m^6^A regulators on chromosomes are shown in Figure 1E. Moreover, we performed principal component analysis (PCA) based on paired tumor-normal specimens and found that the 23 m^6^A regulators completely distinguished CC samples from normal samples (Figure 1F). Further analysis demonstrated that ALKBH5, METTL14, YTHDC2, and YTHDF3 were significantly downregulated in tumor samples, whereas CBLL1, ELAVL1, HNRNPA2B1, HNRNPC, IGF2BP1, KIAA1429, LRPPRC, METTL3, RBM15, and YTHDF1 were significantly upregulated in tumor samples (Figure 1G). The expression of m^6^A regulators with CNV amplification was significantly increased in CC samples compared to normal control samples, such as HNRNPA2B1, IGF2BP1, KIAA1429, and YTHDF1, while METTL14 and YTHDC2 were markedly decreased in the tumor specimens (Figure 1D, 1G). Furthermore, Spearman correlation analysis was performed to evaluate mutual regulation among these m^6^A regulators (Figure S1C). The erasers ALKBH5 and FTO showed a significant inverse correlation with other m^6^A regulators. Cox regression analysis was employed to ascertain the relationship between these m^6^A regulators and the prognosis of CC patients. A forestplot showed that YTHDC1 could be considered a protective factor and was significantly associated with prolonged relapse-free survival, while IGF2BP1 was recognized as a risk factor (Figure S1D-E). The above analysis demonstrated the significant differences and connections in the genomic and transcriptomic landscape of m^6^A regulators between normal and CC samples. Therefore, the expression alterations and genetic variation in m^6^A regulators played a crucial role in regulating CC occurrence and progression.

### Identification of m^6^A methylation modification patterns mediated by 23 regulators

**Figure 2:**
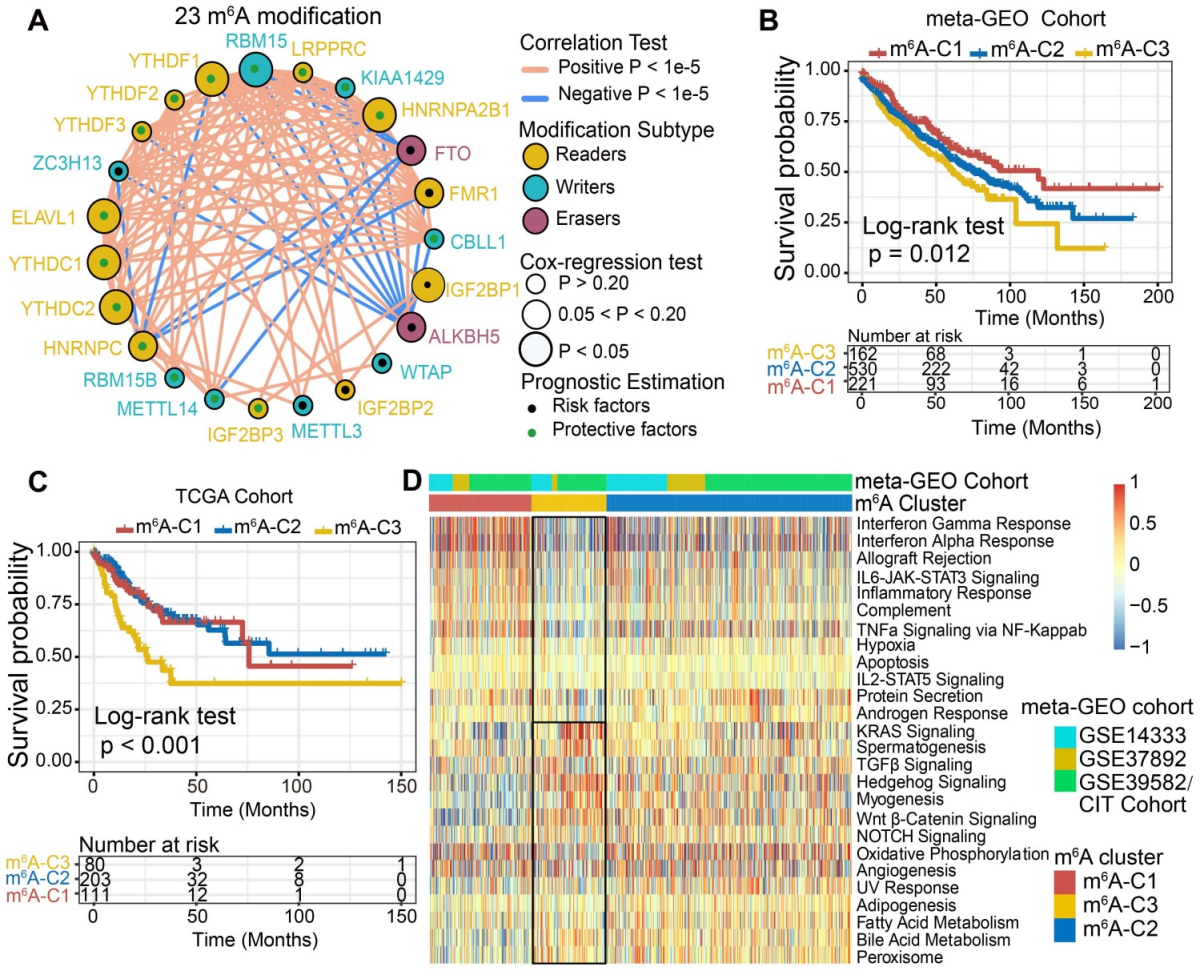
**m^6^A methylation modification pattern and relevant biological pathway.** (A) The interaction of expression on 23 m^6^A regulators in CC. The m^6^A regulators in three RNA modification types were depicted by circles in different colors. Readers, yellow; Writers, blue; Erasers, red. The lines connecting m^6^A regulators represented their interaction with each other. The size of each circle represented the prognosis effect of each regulator and scaled by P-value. Protective factors for patients' survival were indicated by a green dot in the circle center and risk factors indicated by the black dot in the circle center. (B) Kaplan-Meier curves of relapse-free survival (RFS) for 913 CC patients in meta-GEO cohort with different m^6^A clusters. The numbers of patients in m^6^A-C1, m^6^A-C2, and m^6^A-C3 phenotypes are 221, 530, and 162, respectively (Log-rank test). (C) Kaplan-Meier curves of relapse-free survival (RFS) for 394 CC patients in the TCGA cohort with three m^6^A clusters. The numbers of patients in m^6^A-C1, m^6^A-C2, and m^6^A-C3 phenotypes are 111, 203, and 80, respectively (Log-rank test). The m^6^A-C3 showed significantly worse prognostic than the other two m^6^A clusters in both meta-GEO and TCGA-COAD cohorts. (D) Heatmap shows the GSVA score of representative Hallmark pathways curated from MSigDB in distinct m^6^A modification patterns. The GEO cohort composition (GSE14333, GSE37892, and GSE39582) were used as sample annotations.

Three GEO datasets with available survival data and clinical annotations (CIT/GSE39582, GSE14333 and GSE37892) were enrolled in the meta-cohort. The comprehensive landscape of the interactions of the 23 m^6^A regulators, the regulator connections and their prognostic significance in CC patients was illustrated in the m^6^A regulator network (Figure 2A). The results indicated that cross-talk among the regulators of writers, readers and erasers probably plays critical roles in the formation of different m^6^A modification patterns and was implicated in cancer pathogenesis and progression. Based on these hypotheses, we utilized consensus clustering analysis of the NMF algorithm to stratify samples with qualitatively different m^6^A modification patterns based on the expression of 23 m^6^A regulators. Accordingly, we identified three distinct modification pattern clusters, including 221 cases in pattern cluster 1, 530 cases in cluster 2 and 162 cases in cluster 3 (Figure 2B, Figure S2A-C). We termed these clusters m^6^A-C1, m^6^A-C2, and m^6^A-C3, among which m^6^A-C1 exhibited a prominent survival advantage, whereas m^6^A-C3 had the worst prognosis in the meta-GEO cohort (P = 0.012, log-rank test). In addition, we performed identical analyses in the TCGA-COAD cohort, and similar results were obtained (P < 0.001, log-rank test, Figure 2C, Figure S2D). Multivariate Cox proportional hazards regression analysis further demonstrated that this modification model was associated with patient survival outcomes after adjusting for clinicopathologic factors in these two cohorts (meta-GEO cohort: m^6^A-C1* vs.* m^6^A-C3, HR, 0.63 [95%CI, 0.46 to 0.87], P = 0.005, Figure S3A; TCGA-COAD: m^6^A-C1* vs.* m^6^A-C3, HR, 0.49 [95%CI, 0.29 to 0.83], P = 0.008, Figure S3B). We also noticed a significant difference in the expression of m^6^A regulators between distinct m^6^A modification patterns. *IGF2BP1* and *YTHDF1* were significantly elevated in the m^6^A-C3 subtype; *FTO*, *RBM15B*, *METTL14*, and *YTHDC2* were markedly increased in the m^6^A-C2 subtype; and *ALKBH5*, *IGF2BP3* and *YTHDC1* were evidently increased in the m^6^A-C1 subtype (Figure S2C-D).

### The m^6^A modification patterns characterized by distinct immune landscapes

**Figure 3:**
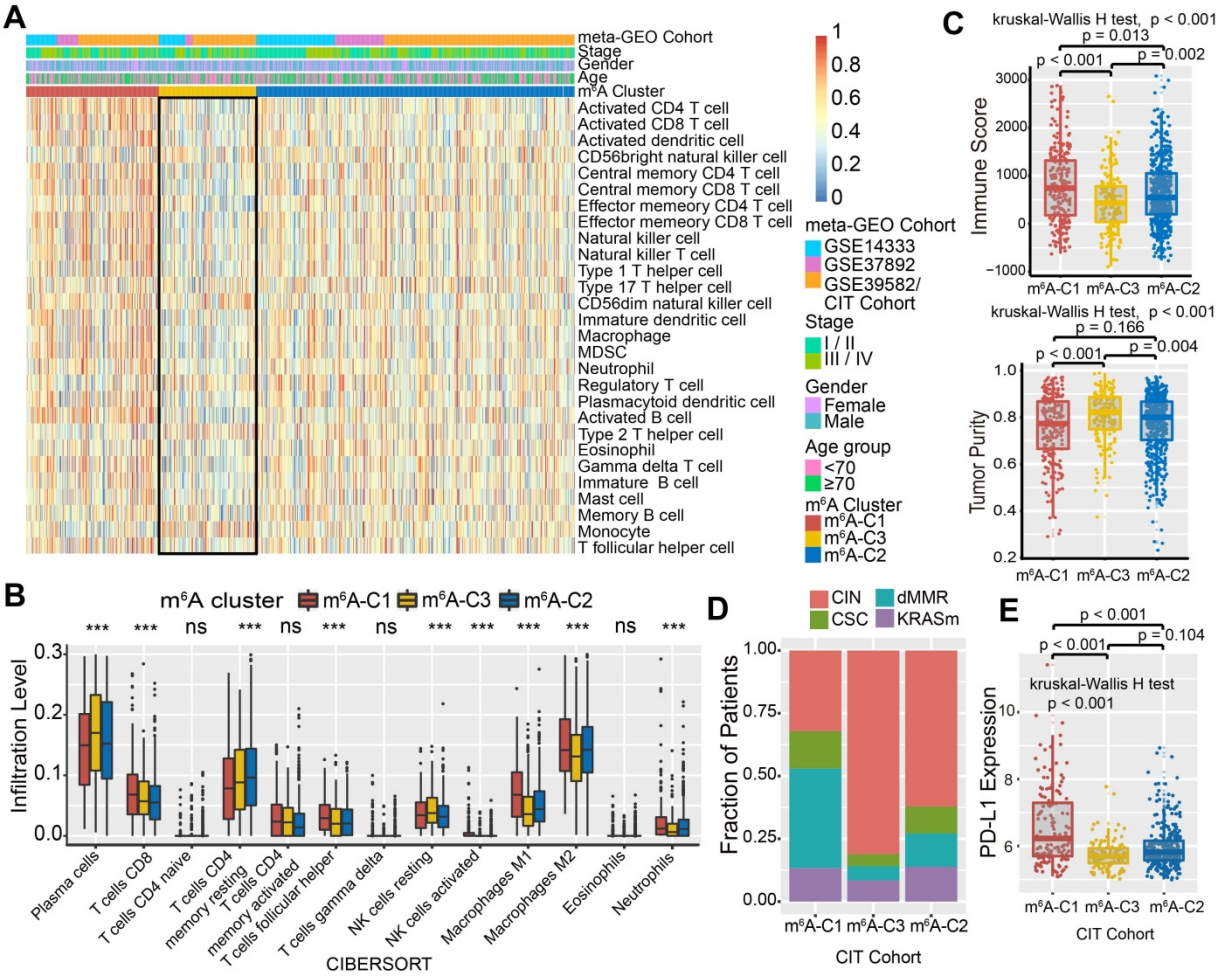
**TME characteristics in distinct m^6^A modification patterns.** (A) Unsupervised clustering of 23 m^6^A regulators in the meta-GEO CC cohort. Clinicopathological information including age, gender, and tumor stage, as well as the m^6^A cluster, is shown in annotations above. Red represented the high expression of regulators and blue represented the low expression. (B) The fraction of tumor-infiltrating lymphocyte cells in three m^6^A clusters using the CIBERSORT algorithm. Within each group, the scattered dots represented TME cell expression values. The thick line represented the median value. The bottom and top of the boxes were the 25th and 75th percentiles (interquartile range). The whiskers encompassed 1.5 times the interquartile range. The statistical difference of three gene clusters was compared through the Kruskal-Wallis H test. *P < 0.05; **P < 0.01; ***P < 0.001. (C) The immune score and tumor purity of three gene clusters were analyzed and plotted. (D) The proportion of molecular subtypes in the three modification patterns. (E) Comparison of PD-L1 expression level across three m^6^A modification patterns.

To explore the biological molecular changes underlying three distinct m^6^A modification patterns, we performed GSVA enrichment analysis against the Hallmarker gene set (Figure 2D). GSVA indicated that m^6^A-C1 was significantly enriched in immune activation-related processes, including interferon gamma/alpha response, allograft rejection and inflammatory response. However, m^6^A-C3 presented enrichment pathways prominently associated with carcinogenic activation and stromal pathways, such as the Wnt-β-catenin, TGF-β, and hedgehog signaling pathways. Intriguingly, m^6^A-C2 was highly enriched in both immune regulation and stromal-related signaling pathways. Furthermore, we constructed a heatmap with ssGSEA to visualize and compare the relative abundances of 28 immune infiltrating cell subpopulations among distinct m^6^A modification patterns (Figure 3A). Antitumor lymphocyte cell subpopulations, such as effector memory CD4^+^/CD8^+^ T cells, activated CD4^+^/CD8^+^ T cells, and NK T cells, were mainly enriched in the m^6^A-C1 and m^6^A-C2 subtypes. However, regulatory T cells, type 2 T helper cells, monocytes, etc. were markedly elevated in the m^6^A-C3 subtype. We also further characterized the immune infiltration profile with CIBERSORT, a deconvolution algorithm using support vector regression to evaluate the immune cell subsets in the TME. A consistent result was also observed in this m^6^A methylation modification stratification (Figure 3B). Previous studies revealed that the immune-excluded tumor phenotype was characterized by an abundance of immune cells, while these immune cells were retained in the stroma surrounding tumor cell nests rather than penetrating their parenchyma 25. Therefore, we speculated that abundant stromal elements in the m^6^A-C2 subtype suppressed an effective antitumor immune response. Subsequent analyses demonstrated that stromal activation was markedly enhanced in the m^6^A-C2 subtype, as exhibited by processes related to epithelial-mesenchymal transition (EMT), TGF-β, and WNT-target pathways, further corroborating our hypothesis (Figure S3C-D). Moreover, we used the ESTIMATE algorithm to quantify the overall infiltration of immune cells (Immune Score) and tumor cell purity (Tumor Purity) across three modification patterns. Further analyses revealed that m^6^A-C1 exhibited the highest immune scores, followed by m^6^A-C2 and m^6^A-C3 (Figure 3C, upper panel). Conversely, m^6^A-C3 had a higher tumor purity than m^6^A-C2 and m^6^A-C1, suggesting that m^6^A-C2 and m^6^A-C1 subtype tumors are surrounded by more nontumor components (e.g., immune cells and stromal cells) (Figure 3C, lower panel). Marisa *et al.* (CIT cohort/GSE39582) stratified CC patients into four dominant molecular subtypes (CIN, CSC, dMMR, and KRASm) and identified dMMR tumors associated with immune coordination while CIN associated with ECM-receptor interaction, focal adhesion, and Wnt receptor pathways 35. Consistent with the previous findings, patients with the CIN subtype were predominantly clustered into m^6^A-C2 and m^6^A-C3, whereas the dMMR subtype was mainly concentrated within m^6^A-C1 tumors (Figure 3D). Considering that PD-L1 is a well-established biomarker for predicting the response to anti-PD-1/L1 treatment 61, we also compared the PD-L1 expression level in different m^6^A modification clusters and observed a significant up-regulation in the m^6^A-C1 subtype (Figure 3E). Based on these findings, we confirmed that the three m^6^A modification patterns were characterized by different immune infiltration profiles. As expected, m^6^A-C1 was recognized as an immune-inflamed phenotype characterized by immune activation and abundant immune cell infiltration; m^6^A-C2 was recognized as an immune-excluded phenotype characterized by stromal activation and weakened immune cell infiltration; and m^6^A-C3 was recognized as an immune-desert phenotype characterized by immune suppression.

We further investigated the specific association between each m^6^A regulator and immune cell infiltration by using Spearman's correlation analyses (Figure S4A). High expression of *ALKBH5*, *FTO*, *IGF2BP3*, and *WTAP* was significantly associated with enhanced immunocyte infiltration, whereas *CBLL1*, *ELAVL1*, *FMR1*, *HNRNPA2B1*, *IGF2BP1*, *KIAA142*9, *LRPPRC*, *RBM15*, *YTHDF1-3*, and *ZC3H13* expression exhibited a negative correlation with the immune infiltration level. Among these m^6^A regulators, the m^6^A binding protein *IGF2BP1* attracted our attention on account of its significant negative correlation with prognostic outcomes and immune infiltration. Moreover, a strong positive correlation between *IGF2BP1* and *YTHDF1* was identified in the aforementioned results (Figure 2A). Current studies revealed the mechanism of m^6^A-regulator *YTHDF1* in mediating the activation of dendritic cells (DCs) and antigen cross-priming of CD8^+^ T cells by enhancing translation of mRNA encoding cathepsins (lysosomal proteases that degrade antigens in phagosomes) 28. Our study also found that *IGF2BP1* exhibited a significant negative correlation with the infiltration levels of activated DCs, immature DCs, and CD8^+^ T cells (Figure S4A-B). We next stratified *IGF2BP1* into high versus low expression subgroups and explored the molecular pathogenesis behind *IGF2BP1*. GSEA analysis demonstrated that samples with low *IGF2BP1* expression showed enrichment of genes involved in the interferon gamma/alpha response, TNFα via NF-κB, and inflammatory response signaling pathways (Figure S4C). The ESTIMATE algorithm exhibited a higher immune score in the *IGF2BP1* low-expression subgroup, which confirmed the above findings (Figure S4D). Four categories of immunogenicity analysis revealed that the low *IGF2BP1* expression subgroup was characterized by the upregulation of cross-presentation-related MHC molecules and immune effector cells but downregulation of immune suppressor cells (Figure S4E). In addition, we observed that tumors with *IGF2BP1* mutations harbored significantly greater TML than those without *IGF2BP1* mutations (Figure S4F). Taken together, we speculated that *IGF2BP1* may coordinate with *YTHDF1* to mediate methylation modification that suppressed the activation of DCs and cytotoxic T lymphocytes, thus impeding the intratumoral antitumor immune response.

### m^6^A phenotype-related DEGs in colon cancer

**Figure 4:**
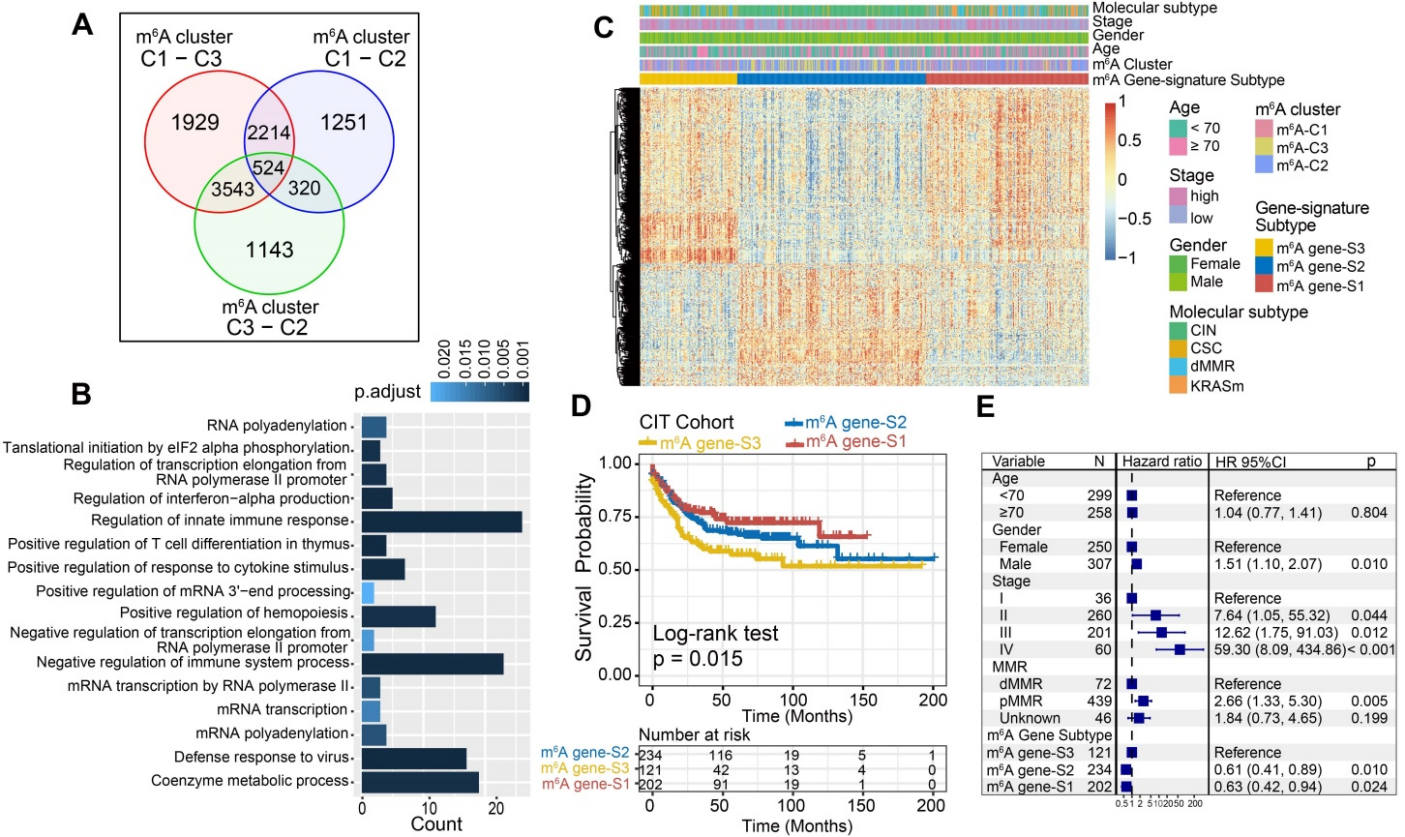
** Construction of differential expression of m^6^A gene signatures and functional annotation.** (A) 524 m^6^A-related differentially expressed genes (DEGs) between three m^6^A-clusters were shown in the Venn diagram. (B) Functional annotation for m^6^A-related genes using GO enrichment analysis. The color depth of the barplots represented the number of genes enriched. (C) Unsupervised clustering of overlapping m^6^A phenotype-related DEGs to classify patients into different genomic subtypes, termed as m^6^A gene S1-3, respectively. The gene signature subtypes, m^6^A clusters, molecular subtypes, tumor stage, gender, and age were used as patient annotations. (D) The survival curves of the m^6^A phenotype-related gene signatures were estimated by the Kaplan-Meier plotter. (P = 0.015, Log-rank test). (E) Subgroup analysis estimating clinical prognostic value between m^6^A gene signature in independent CC data sets and cancer stage by univariate Cox regression. The length of the horizontal line represented the 95% confidence interval for each group. The vertical dotted line represented the hazard ratio (HR) of all patients.

Although the consensus clustering algorithm based on m^6^A regulator expression classified CC patients into three m^6^A modification phenotypes, the underlying genetic alterations and expression perturbations within these phenotypes were not well known. Based on these queries, we further examined the potential m^6^A-related transcriptional expression change across three m^6^A modification patterns in CC. The empirical Bayesian approach was applied to determine overlapping differentially expressed genes (DEGs) among the three m^6^A modification patterns. A total of 524 DEGs that represented the critical distinguishing index of the three m^6^A modification patterns were considered as m^6^A-related signature and illustrated in a Venn diagram (Figure 4A, Table S5). GO enrichment analysis of these signature genes revealed that the biological processes related to RNA modification, transcription, and immune regulation were significant over-represented (Figure 4B). These results further demonstrated the overlapped DEGs were characterized by m^6^A modification and immunity, and could be regarded as the m^6^A-related gene signatures. Based on the 524 most representative m^6^A phenotype-related signature genes, we performed unsupervised consensus clustering analysis and obtained three stable transcriptomic phenotypes (Figure S5A-B). These stratifications divided patients into three distinct m^6^A gene signature subgroups that had different clinicopathologic features and were defined as m^6^A gene-S1, m^6^A gene-S2 and m^6^A gene-S3 (Figure 4C). We found that patients with an advanced clinical stage were represented by the m^6^A gene-S3 subgroup, and patients with CIN subtypes and down-regulated PD-L1 expression were mainly concentrated in the m^6^A gene-S2 and S3 subgroups (Figure 4C, Figure S5C). Further survival analysis indicated a significant prognostic differences among the three m^6^A gene signatures in CC samples. The m^6^A gene-S1 signature was proven to be associated with better prognosis, while m^6^A gene-S3 was associated with worse survival outcomes (Figure 4D). The association between the m^6^A gene signatures with survival remained statistically significant after considering age, gender, MMR and stage (Cox proportional hazards model, m^6^A-S2 *vs.* m^6^A-S3, HR, 0.61 [95%CI, 0.41 to 0.89], P = 0.010; Figure 4E). The expression levels of the 23 m^6^A regulators among three gene signature subgroups were also compared and shown in Figure S5D. We observed significant differences in m^6^A regulator expression between the three m^6^A gene signature subgroups, which was consistent with the expected results of the m^6^A methylation modification patterns.

### Construction of the m^6^Sig score and exploration of its clinical relevance

**Figure 5:**
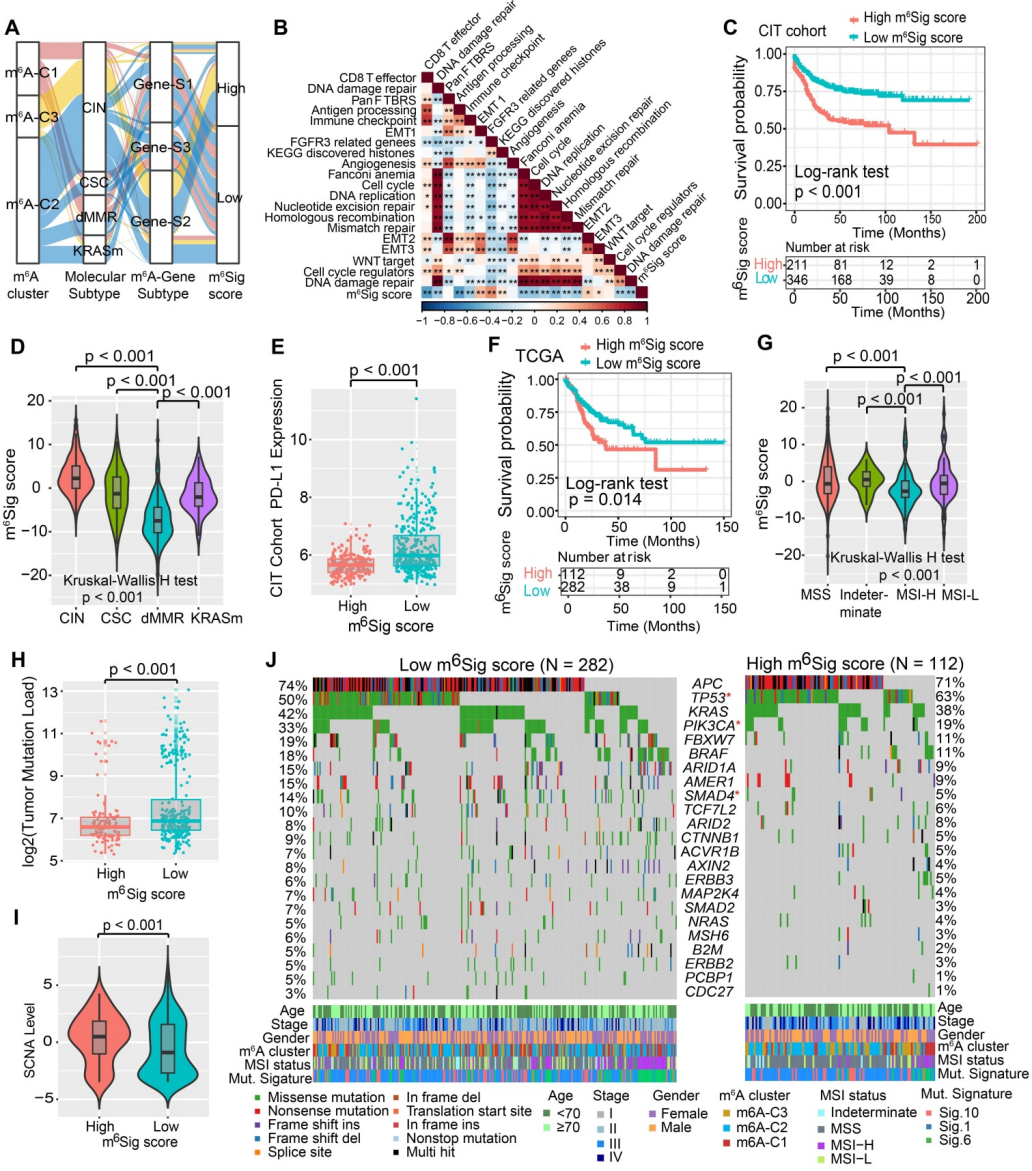
** Construction of m^6^Sig score and explore the relevance of clinical features.** (A) Alluvial diagram of m^6^A clusters in groups with different molecular subtypes (CIN, CSC, dMMR and KRASm), m^6^A-gene cluster, and m^6^Sig score. (B) Correlations between m^6^Sig score and the known biological gene signatures using Spearman analysis. The negative correlation was marked with blue and positive correlation with red. (C) Kaplan-Meier curves for high and low m^6^Sig score patient groups in CIT cohort. Log-rank test, P < 0.001. (D) Distribution of m^6^Sig score in the different molecular subtypes. The thick line represented the median value. The bottom and top of the boxes were the 25th and 75th percentiles (interquartile range). The whiskers encompassed 1.5 times the interquartile range. The differences between every two groups were compared through the Kruskal-Wallis H test. (E) Relative distribution of PD-L1 expression in high m^6^Sig score versus low m^6^Sig score subgroups. (F) Kaplan-Meier curves for patients with high and low m^6^Sig score subgroups in the TCGA cohort. (G) Violin plot showing m^6^Sig score in groups with high or low MSI and stable status. The differences between the four groups were compared through the Kruskal-Wallis test. MSS, microsatellite stable; MSI-H, high microsatellite instability; MSI-L, low microsatellite instability. (H-I) Relative distribution of tumor mutation load (H) and somatic copy number alternation (I) in m^6^Sig score high versus low subgroups. (J) Mutational landscape of SMGs in TCGA-COAD stratified by low (left panel) versus high m^6^Sig score (right panel) subgroups. Individual patients were represented in each column. The upper barplot showed TML, the right bar plot showed the mutation frequency of each gene in separate m^6^Sig score groups. m^6^A cluster, stage, gender, MSI status, and mutational signatures were shown as patient annotations.

Although our findings identified the role of m^6^A modification in prognosis and immune infiltration modulation, these analyses were only based on the patient population and could not accurately predict the patterns of m^6^A methylation modification in individual tumors. Therefore, we developed a scoring scheme termed the m^6^Sig score, which is based on the identified m^6^A-related signature genes, to quantify the m^6^A modification pattern of individual CC patients. Considering the complexity of the quantification of m^6^A modification, we illustrated the workflow of m^6^Sig score construction with the alluvial diagram (Figure 5A). These results indicated that m^6^A gene-S2 with the CIN subtype was linked to a higher m^6^Sig score, whereas m^6^A gene-S1 exhibited a lower m^6^Sig score. Notably, m^6^A-C3 showed the highest m^6^Sig score, followed by m^6^A-C2 and m^6^A-C1 (Figure S6A). We examined the relationship between known biological signatures and the m^6^Sig score through Spearman analysis. A heatmap of the correlation matrix demonstrated that the m^6^Sig score was markedly negatively correlated with the immune activation process and DNA repair signatures but positively correlated with EMT and stromal-relevant signatures (Figure 5B). There was also a significant inverse correlation between the m^6^Sig score and the immune score (r = -0.44, P < 0.001), demonstrating the crosstalk between the m^6^Sig score and immune infiltration evaluation (Figure S6B). Similarly, compared with the subgroups of patients with a high m^6^Sig score, the low m^6^Sig score subgroup had higher proportions of MHC molecules and immune effector cells but lower proportions of suppressor cells and checkpoint molecules (Figure S6C). Furthermore, we sought to determine the prognostic ability of the m^6^Sig score in predicting survival outcome by dividing the patients into low- or high-score subgroups with a cutoff value of 1.8947 (Methods section). As expected, patients with low m^6^Sig score were significantly associated with a better prognosis in the CIT cohort (HR, 0.47 [95%CI, 0.35 to 0.63], P < 0.001, Figure 5C), and the results of the ROC curves analysis validated the predictive advantage of the established risk model (AUC = 0.732, Figure S6D). Furthermore, multivariate Cox regression model analysis considering patient age, gender, tumor stage, molecular subtype and MSI status confirmed the m^6^Sig score as a robust and independent prognostic biomarker for evaluating patient outcomes (HR, 0.58 [95%CI, 0.42 to 0.80], P < 0.001, Figure S6F). We also explored the relationship between the m^6^Sig score and molecular subtype and found that the dMMR subtype was associated with a lower m^6^Sig score than other CC subtypes (Figure 5D). Additionally, the PD-L1 expression level was investigated, and there was a pronounced elevation in the low m^6^Sig score group (Figure 5E).

We next validated the constructed m^6^A scoring system by integrating the clinical characteristics and genomic information of TCGA-COAD database. It was also found that m^6^Sig score displayed the potential prognosis predictive value in TCGA cohort (AUC = 0.704, Figure S6E), and patients with low m^6^Sig score indicated a prominent survival benefit (HR, 0.62 [95%CI, 0.42 to 0.91], P = 0.014; Figure 5F). Multivariate analysis for the TCGA cohort also confirmed that the m^6^Sig score could act as an independent prognostic biomarker in CC (HR, 0.65 [95%CI, 0.41 to 0.90], P = 0.009; Figure S6G). TCGA analysis revealed a comprehensive molecular characterization of CC and suggested subdividing tumors into four subtypes based on MSI status. A significant lower m^6^Sig score was found in samples of the MSI-H subtype than in those of the other three subtypes (Figure 5G). To further verify the reliability of the m^6^A scoring model, we utilized the two aforementioned CC cohorts and an independent cohort to determine the relationship between the m^6^Sig score and patient prognosis (GSE14333, GSE37892 and GSE33113). Consistent with the above findings, the low m^6^Sig score group showed an obvious survival advantage over the high score group (Figure S7A-C). The above results strongly suggested that the m^6^Sig score can represent the m^6^A modification patterns and predict the prognosis of CC patients.

Increasing evidence has demonstrated an association between the tumor genome somatic mutations and responsiveness to immunotherapy. Consequently, we investigated the distribution patterns of tumor mutation load in different m^6^Sig score groups and revealed that the low m^6^Sig score group had higher mutation frequencies (Figure 5H). We also noticed a higher frequency of somatic copy number alterations (SCNA) in the low m^6^Sig score subgroup, consistent with the previous finding that SCNA correlated positively with immune evasion and tumor cell proliferation (Figure 5I). We further performed significantly mutated gene (SMG) analysis for CC samples in the low m^6^Sig score subgroup versus the high score subgroup. The SMG mutational landscapes showed that *PIK3CA* (33% *vs.* 19%) and *SMAD4* (14% *vs.* 5%) had higher somatic mutation rates in the low m^6^Sig score group, whereas *TP53* (50%* vs.* 63%) had higher somatic mutation rates in the high m^6^Sig score subtype (Fisher's exact test, P < 0.05, Figure 5J). To gain further insights into the mutational processes operative in CC, we extracted three mutational signatures (known as signatures 1, 6, and 10) from the TCGA-COAD mutational profile. We found that the low m^6^Sig score subtype exhibited a significantly higher proportion of mutational signature 6 (Fisher's exact test, P = 0.013, Figure 5J). These data enabled us to depict the effect of m^6^Sig score classification on genomic variation more comprehensively, as well as to reveal the potentially complex interaction between individual somatic mutations and m^6^A modifications.

### The role of m^6^Sig score in predicting immunotherapeutic benefits

**Figure 6:**
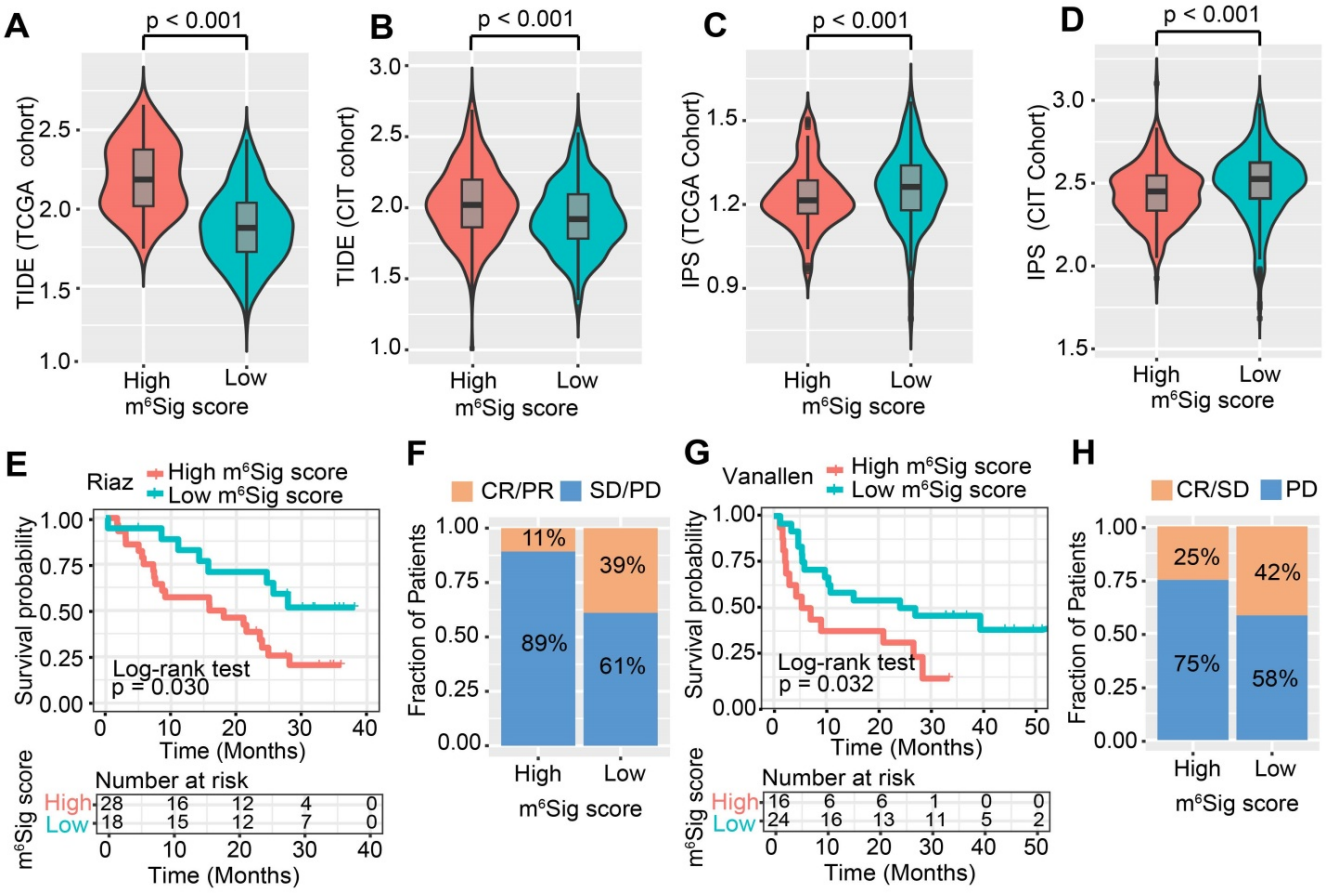
** The m^6^Sig score predicts immunotherapeutic benefits.** (A-D) The relative distribution of TIDE was compared between m^6^Sig score high versus low groups in TCGA-COAD (A) and CIT (B) cohort, respectively. The relative distribution of IPS was also compared between m^6^Sig score high and low groups in TCGA-COAD (C) and CIT (D) cohort. (E) Kaplan-Meier curves for high and low m^6^Sig score patient groups in the Riaz *et al.* cohort. Log-rank test, P = 0.030. (F) The fraction of patients with clinical response to anti-PD-1 immunotherapy (Riaz *et al.* cohort) in low or high m^6^Sig score groups. CR/PR *vs.* SD/PD: 39%* vs.* 61% in the low m^6^Sig score groups, 11%* vs.* 89% in the high m^6^Sig score groups. (G) Kaplan-Meier curves for high and low m^6^Sig score patient groups in the Vanallen *et al.* cohort. Log-rank test, P = 0.032. (H) The fraction of patients with clinical response to anti-CTLA-4 immunotherapy in low or high m^6^Sig score groups of Vanallen *et al.* cohort. CR/SD *vs.* PD: 42% *vs.* 58% in the low m^6^Sig score groups and 25% *vs.* 75% in the high m^6^Sig score groups. CR, complete response; PR, partial response; SD, stable disease; PD, progressive disease.

ICI treatment represented by CTLA-4/PD-1 inhibitors has undoubtedly caused a major breakthrough in antitumor therapy. In addition to well-known TML, PD-L1, and MSI 48, 62, newly identified predictors, such as TIDE and IPS, are widely used and strongly recommended to evaluate the immune response. Our analysis also revealed that the TIDE was significantly decreased in the low m^6^Sig score group, and IPS was significantly elevated in the low m^6^Sig score group (TIDE distribution in TCGA-COAD and CIT, both P < 0.001, Figure 6A-B; IPS distribution in TCGA-COAD and CIT, both P < 0.001, Figure 6C-D). These findings indirectly demonstrated that the representation of tumor m^6^A modification patterns plays a crucial role in mediating the immune response.

Considering the strong connection of the m^6^Sig score with the immune response, we next investigated whether the m^6^A modification signature could predict patients' response to ICI therapy in three independent immunotherapy cohorts. Firstly, in both the anti-PD-1 cohort 57 (Riaz *et al.* study) and anti-CTLA-4 cohort 58 (Vanallen *et al.* study), patients with a low m^6^Sig score group exhibited significant clinical advantages and markedly prolonged survival (anti-PD-1, HR, 0.40 [95%CI, 0.18 to 0.92], P = 0.030. Figure 6E; an-CTLA-4, HR, 0.42 [95%CI, 0.19 to 0.92], P = 0.032, Figure 6G). The significant therapeutic benefits and immune response to ICI treatment were confirmed in patients with low m^6^Sig score compared to those with high m^6^Sig score (response rate of anti-PD-1 cohort: 39% *vs.* 11%, Figure 6F; response rate of anti-CTLA-4 cohort: 42% *vs.* 25%, Figure 6H). A consistent result was also observed in the anti-PD-L1 cohort (Mariathasan *et al.* study), lower m^6^Sig scores in mUC patients were significantly associated with better clinical outcomes and higher tumor mutational load (anti-PD-L1, HR, 0.66 [95%CI, 0.49 to 0.88], P = 0.004, Figure S7D-F). Taken together, our findings strongly suggest that the m^6^Sig score is associated with the response to immunotherapies and can further predict the prognosis of patients.

## Discussion

Mounting evidence has shown that m^6^A modification plays an essential role in innate immunity, inflammation, and antitumor effects through interaction with diverse m^6^A regulators 6, 7, 63. Although plenty of elegant studies have revealed the epigenetic modulation of m^6^A regulators in the immune contexture, the overall TME characteristics mediated by integrated m^6^A regulators have not been comprehensively recognized. Therefore, identifying distinct m^6^A modification patterns in the tumor immune microenvironment will provide insights into the interactions of m^6^A RNA methylation on anti-tumor immune response and facilitate more effective precision immunotherapy strategies.

In this study, we identified three distinct m^6^A methylation modification patterns characterized by different immune phenotypes, which were correlated with diverse anticancer immunity. The m^6^A-C1 was characterized by immune activation and tumor-infiltrating lymphocyte infiltration, corresponding to an immune-inflamed phenotype. The m^6^A-C2 was characterized by the presence of immune cells and stroma, together with EMT, TGF-β and Wnt signaling pathway activation, corresponding to an immune-excluded phenotype. The m^6^A-C3 was characterized by the immunosuppression TME, corresponding to an immune-desert phenotype. A previous study demonstrated that the tumor microenvironment contexture plays a crucial role in tumor progression and immunotherapeutic efficacy 20. Baseline levels of tumor-infiltrating CD4^+^/CD8^+^ T cells, Macrophage M1, NK cells and inflammatory cytokines secretion *et al.* have been shown to be correlated with the likelihood of immune response 20, 64, 65. We also identified that the m^6^A-C1 pattern was significantly associated with elevated tumor-infiltrating lymphocyte and PD-L1 levels, supporting the potential predictive value on immunotherapy benefits. Recent studies reported that the activation of EMT- and TGF-β-related pathways impeded the penetration of lymphocyte cells into the parenchyma of these tumors 66. Specific molecular inhibitors targeting TGF-β have been shown to reshape the tumor microenvironment (e.g. reprogram peritumoral stromal fibroblasts) and restore the anti-tumor immunity 27, 67. Based on these findings, we speculated that CC patients with the m^6^A-C2 pattern may benefit from combination treatment with ICB agents and TGF-β blockade.

Furthermore, differentially expressed genes (DEGs) identified from distinct m^6^A modification patterns were significantly over-represented in biological pathways implicated in RNA polyadenylation and immunity, suggesting that these DEGs were considered as m^6^A phenotype-related gene signatures. Similar to the results of m^6^A modification clustering, three transcriptomic subtypes based on m^6^A signature genes were identified and were significantly associated with different survival outcomes and TME landscapes. We further established a quantification system named the 'm^6^Sig score' to define different m^6^A modification patterns, thus guiding therapeutic strategies for individual patients more precisely. As a result, the m^6^A modification pattern characterized by the immune-excluded phenotype and immune-desert phenotype exhibited a higher m^6^Sig score, while the pattern characterized by the immune-inflamed phenotype showed a lower m^6^Sig score. Further analyses highlighted that the m^6^Sig score was a prognostic biomarker in colon cancer and associated with mutational signatures, SCNA and MSI-H status, suggesting that the m^6^Sig score may serve as a preferable surrogate for genomic aberrations. In addition, we observed that the m^6^Sig score was strongly associated with predictors of the immune response, including TML, PD-L1, IPS, and TIDE, implying that m^6^A modification could influence the therapeutic efficacy of immunotherapy. Actually, we identified the robust prediction ability of the m^6^Sig score in the immune response via three independent ICI cohorts. These findings verified our hypothesis that the m^6^A modification pattern could be applied in clinical practice to determine immune phenotypes and guide therapeutic regimens.

Besides elucidated the clustering results of m^6^A-modification, we also explored the specific role of individual m^6^A-regulator in the regulation of tumor immunity. Recent advances have indicated that RNA N^6^-methyladenosine enhances mRNA stability and translation mainly through mRNA binding proteins of IGF2BPs 68. Of these, *IGF2BP1* was recognized as a tumor oncogene as it impairs miRNA-directed downregulation of oncogenic factors in various cancer types 69, 70. Our analyses revealed that *IGF2BP1* was up-regulated in tumor tissues and associated with reduced survival time. Further analysis indicated a strong positive correlation between* IGF2BP1* and *YTHDF1* in colon cancer. A previous study reported that *YTHDF1* could recognize m^6^A-marked transcripts encoding lysosomal proteases and result in degradation of neoantigens in dendritic cells, thereby suppressing cross-presentation of tumor neoantigens and cross-priming of CD8^+^ T cells 28. Furthermore, higher expression of *IGF2BP*1 exhibited a significantly lower infiltration level of activated DCs and immature DCs, suggesting that *IGF2BP1* mediated m^6^A modification may be involved in the activation of TME DCs. *IGF2BP2/3* were also functioned as 'readers' in identifying and stabilizing the m^6^A site, and played a nonnegligible role in colorectal carcinoma progression via post-transcriptional regulation manner 71, 72. Although high expression of *IGF2BP2* was not a strong indicator of prognosis, leukocyte subsets analyses indicated that it was significantly negatively correlated with the infiltration of tumor infiltrating lymphocytes (TILs). Accordingly, the m^6^A modification effect of *IGF2BP1/2* on immunosuppressive mechanisms still require further validation in low-throughput biological experiments with cell culture and PDX mouse models. *KIAA1429* and *RBM15/15B* contain RNA-binding domains, and thus may facilitate the recruitment of the 'writer' complex to specific sites in mRNA 73, 74. Here, we observed that* KIAA1429* and *RBM15* had prevalent CNV alterations and significant up-regulation in tumor tissues, suggesting the potential role of promoting the migration and invasion of cancer cells 75. Subsequent correlation analysis also revealed that the two m^6^A-writers were inversely associated with immune cell infiltration. Inversely, the m^6^A RNA demethylase *ALKBH5* exhibited an obvious enhancement in T lymphocyte infiltration and significant up-regulation in normal samples, indicating that the multiple types of post-transcriptional regulation underlay anti-tumor effects 76, 77. Together, these results suggest a diverse heterogeneity of m^6^A modification, demonstrating the importance of a comprehensive assessment of the m^6^A modification patterns and enhancing our understanding of epigenetic regulation on diverse physiological processes.

Evaluation of the mutated driver genes underlying human tumors is a critical foundation for cancer diagnostics, therapeutics, and rational therapy selection. Here, we identified that the mutation rates of the SMGs of *PIK3CA* and *SMAD4* were markedly augmented in the low score subgroup compared to the high score subgroup, while the *TP53* mutation rate was elevated in the high score subgroup. Previous studies demonstrated that the *PIK3CA* mutation was associated with increased immune cell infiltration and decreased tumoral PD-L1 expression 78, 79. *SMAD4* belongs to the *SMAD* protein family, which is involved in the TGF-β signaling pathway that usually impedes immune activation in the tumor microenvironment 80. *TP53* is frequently mutated in most tumor types, and its mutation results in the downregulation of the immune response in hepatocellular carcinoma 81. These m^6^Sig score-related tumor driver gene mutations were markedly associated with the immune activity, suggesting the complicated interaction of m^6^A modification with tumor immunogenomic characteristics.

Although we reviewed the literature and curated a catalog of 23 recognized regulators of RNA methylation, a series of new identified regulators need to be incorporated into the model to optimize the accuracy of the m^6^A modification patterns. In the absence of an appropriate ICI-based colon cancer dataset, we hope that combined with different immunotherapy regimens (anti-PD1/L1 or anti-CTLA-4) across different malignancies (Melanoma and Urothelial cancer) to verify the effects of m^6^Sig score could further strengthen our conclusion. Besides, the m^6^A modification patterns and m^6^Sig score were identified by using retrospective datasets; thus, a prospective cohort of CC patients receiving immunotherapy is needed to validate our findings. Moreover, as not all patients with low m^6^Sig score exhibited robust clinical benefits from ICI therapy, more clinicopathological features should be incorporated into the prediction models to improve accuracy.

In this study, we comprehensively evaluated the m^6^A modification patterns among 1307 colon cancer samples based on 23 m^6^A regulators, and systematically correlated these modification patterns with TME cell-infiltrating characteristics. This integrated analysis indicated that dysregulation of RNA methylation lays a critical foundation for understanding the regulation of tumor immunity. More broadly, evaluating the m^6^A modification patterns of the individual tumor will contribute to enhancing our cognition of the characteristics of TME infiltration and provide important insight into immunotherapeutic efficacy.

## Supplementary Material

Supplementary figures.

Supplementary tables.

## Abbreviations

CC: Colon cancer
NMF: nonnegative matrix factorization
PCA: principal component analysis
ICI: immune check-point inhibitors
RFS: relapse-free survival
TME: tumor microenvironment
TML: tumor mutation load
HR: hazards ratio
OR: odds ratio
GSEA: gene set enrichment analysis
COAD: Colon adenocarcinoma
MSI: microsatellite instability
dMMR: Mismatch repair deficiency
IPS: Immunophenoscore
TIDE: Tumor Immune Dysfunction and Exclusion
