# Supplementary material for: m6A regulator-based methylation modification patterns characterized by distinct tumor microenvironment immune profiles in colon cancer
Source: Theranostics. 2021 Jan 1;11(5):2201–17. doi: 10.7150/thno.52717 (PMC7797678; doi:10.7150/thno.52717)
Supplement: Supplementary file 1 — Supplementary figures. [file thnov11p2201s1.pdf]

Supplementary files

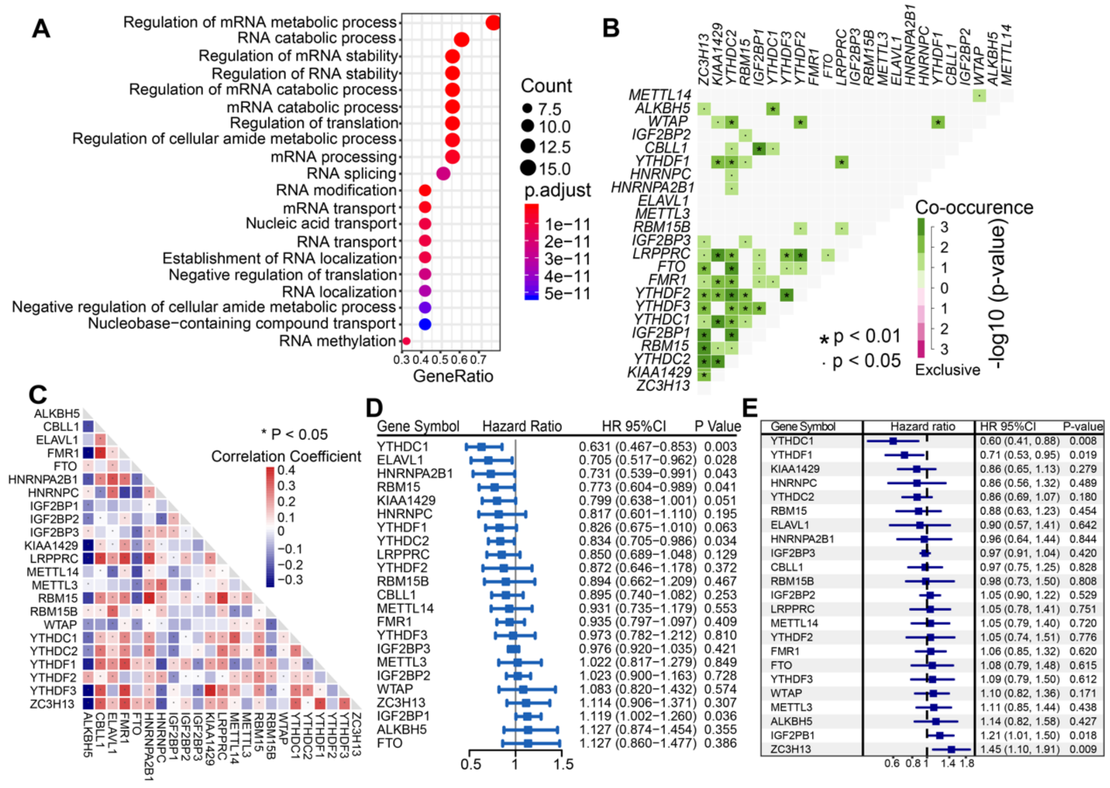

Figure S1. Correlation and prognostic analysis of 23 m^{6}A regulators. (A) GO enrichment analysis of the 23 m^{6}A regulators. The x-axis indicated gene ratio within each GO term. (B) The mutation co-occurrence and exclusion analysis for 23 m^{6}A regulators. Co-occurrence, green; Exclusion, purple. (C) The correlations between these m^{6}A regulators were calculated in CC using the Spearman correlation analysis. The negative correlation was marked with blue and positive correlation with red (*P < 0.05). (D-E) Subgroup analysis estimating clinical prognostic significance between m^{6}A regulators by univariate Cox regression (D) and multivariate Cox regression models (E). The length of the horizontal line represented the 95% confidence interval for each group. The vertical dotted line represented the hazard ratio (HR) of all patients. The vertical solid line represented HR = 1. Hazard ratio >1 represented risk factor for survival and hazard ratio < 1 represented protective factor for survival.

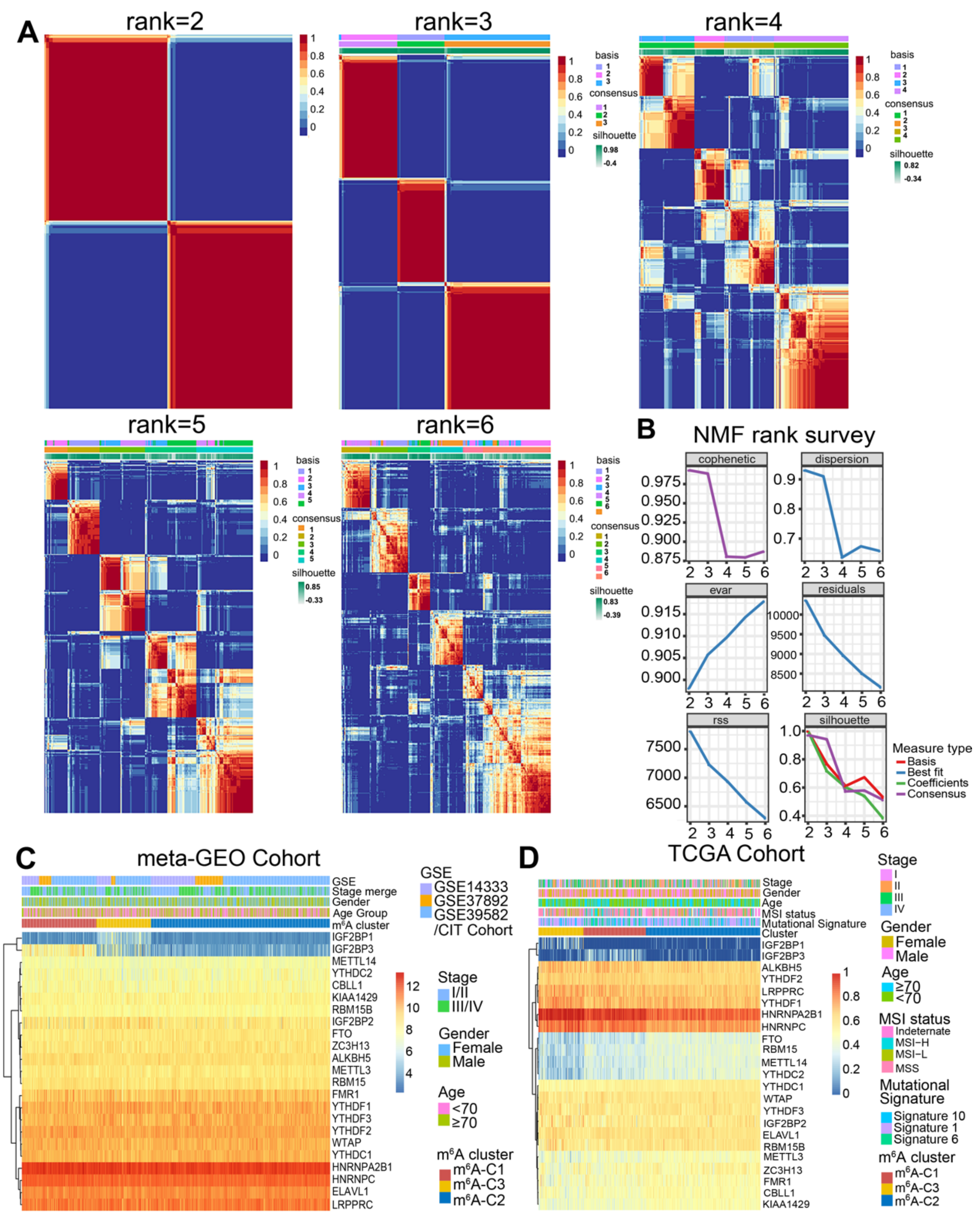

Figure S2. Unsupervised clustering of 23 m^{6}A regulators in the meta-GEO and TCGA cohort. (A) Heatmap representation of NMF clustering for m^{6}A regulators in meta-GEO with cluster numbers from 2 to 6. (B) The relationship between cophenetic, dispersion, residuals, and silhouette coefficients with respect to the number of clusters. (C-D) Unsupervised clustering of 23 m^{6}A regulators in the meta-GEO CC cohort (C) and TCGA cohort (D). The m^{6}A cluster, gender, tumor stage, and age were used as patient annotations. Red represented the high expression of regulators and blue

represented low expression.

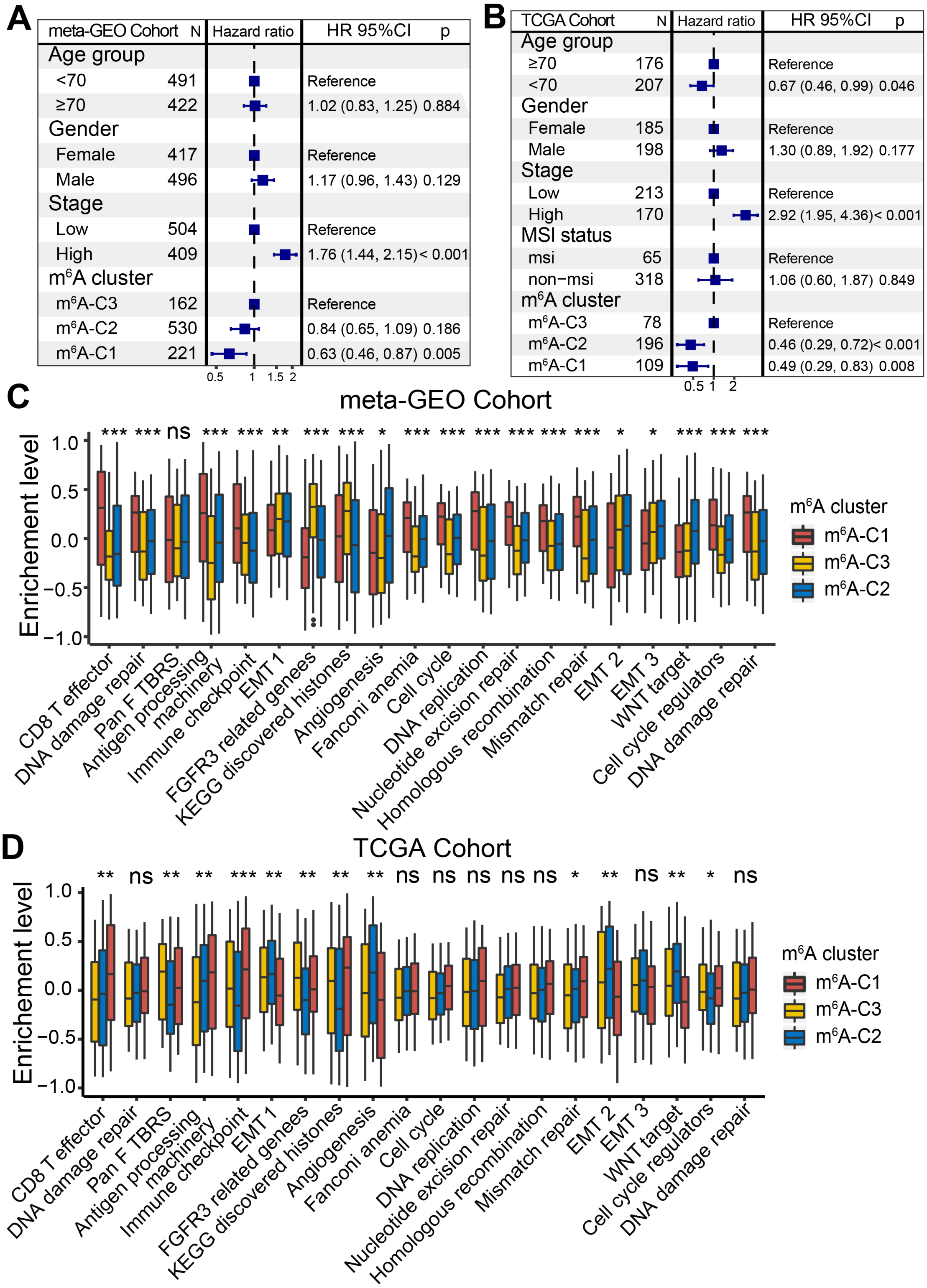

Figure S3. The prognostic value of m^{6}A clusters and enrichment of different signatures in three m^{6}A modification patterns. (A-B) Subgroup analysis estimating clinical prognostic value between m^{6}A clusters in the meta-GEO cohort (A) and TCGA

cohort (B) by multivariate Cox regression. The length of the horizontal line represented the 95% confidence interval for each group. The vertical dotted line represented the hazard ratio (HR) of all patients. m^{6}A clusters were distinguished by different signatures (immune-relevant signature, DNA repair-relevant signature, and stromal-relevant signature as indicated) in the meta-GEO (C) and TCGA (D) cohort. Statistical significance: *P < 0.05; **P < 0.01; ***P < 0.001.

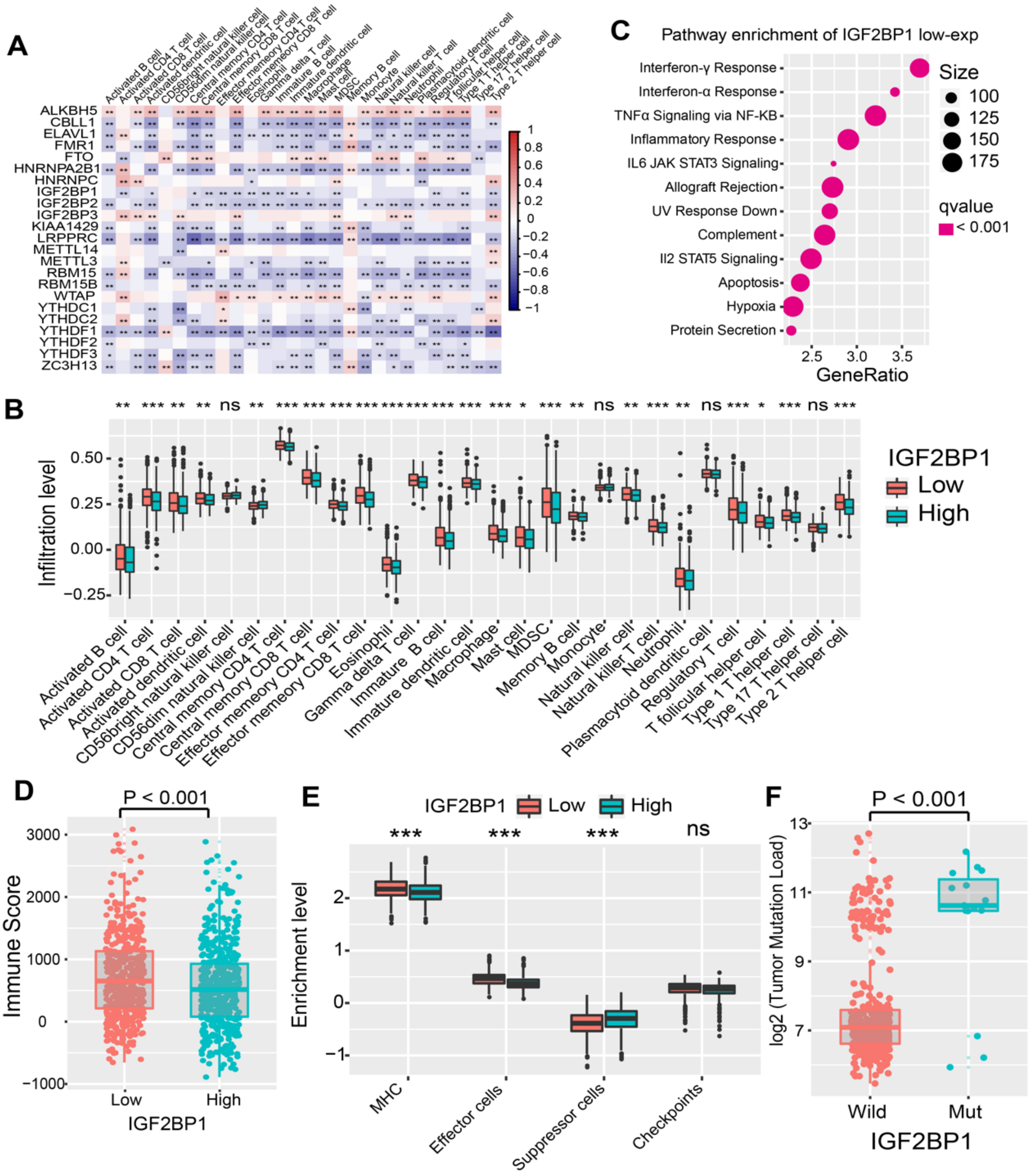

Figure S4. Correlation between TME infiltration and m^{6}A regulators and the roles of *IGF2BP1* in colon cancer. (A) The correlation between each TME infiltration cell type and each m^{6}A regulator using spearman analysis. (B) The difference in the

abundance of each TME infiltrating cell subpopulation between *IGF2BP1* high and low expression group. (C) GSEA pathway enrichment of the top differentially expressed gene sets in *IGF2BP1* low versus high expression group. The cutoff value was based on the median expression of *IGF2BP1*. The degree of color represented q value and the size of node represented the gene number in this item. (D) Distribution of Immune Score in different *IGF2BP1* expression subgroups. (E) Differences in major parameters of determining immunogenicity between *IGF2BP1* high and low expression groups. MHC: MHC class I, class II, and nonclassical molecules; Effector cells: activated $CD8^+$ T cells and $CD4^+$ T cells, Tem $CD8^+$ and Tem $CD4^+$ cells; Suppressor cells: Tregs and MDSCs; Checkpoints: immunoinhibitors and immunostimulators. (F) Relative tumor mutation burden in *IGF2BP1* wild and mutant-type group. Statistical significance: *P < 0.05; **P < 0.01; ***P < 0.001.

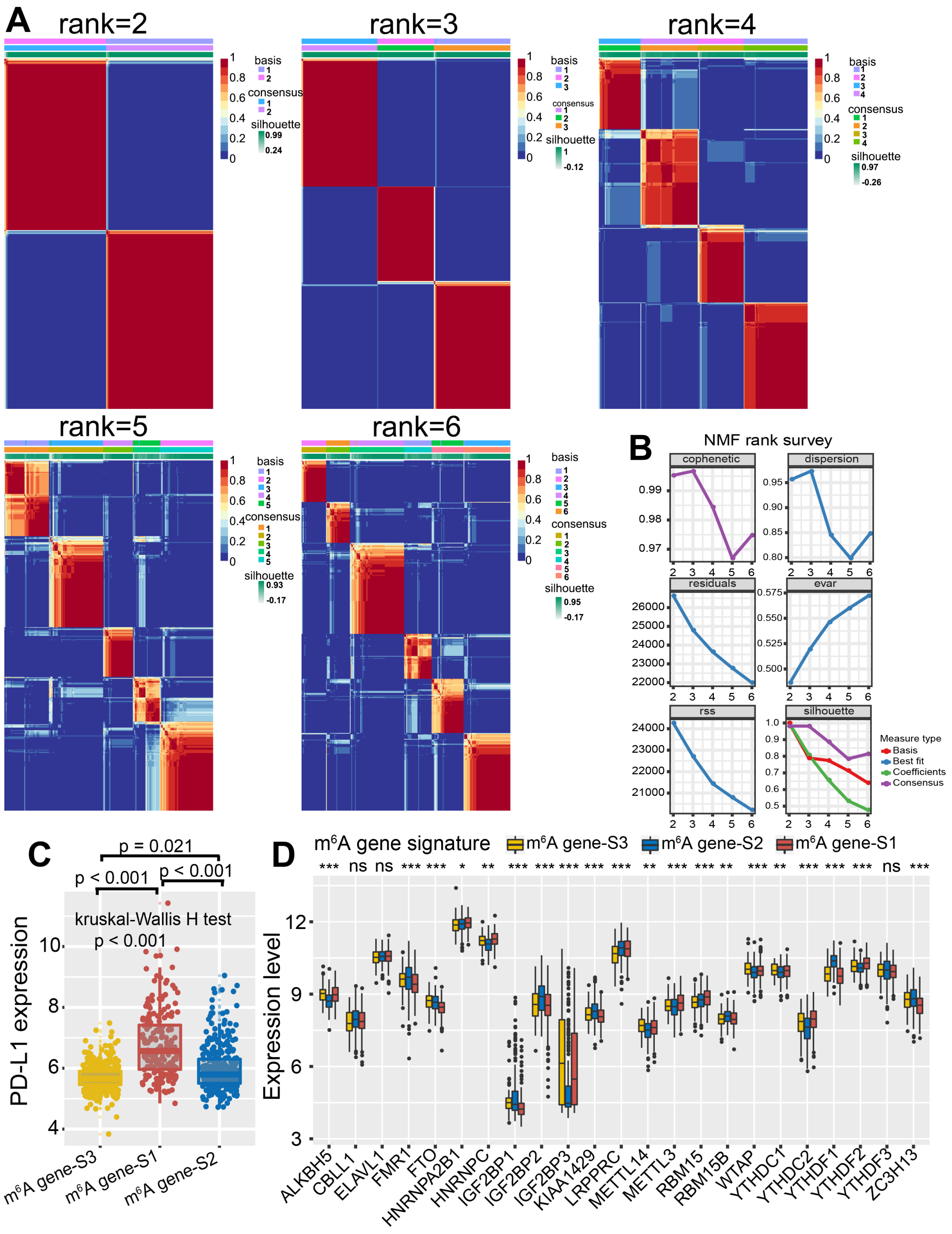

Figure S5. Unsupervised clustering representation of 524 m^{6}A phenotype-related genes in the colon cancer cohort. (A) Heatmap representation of NMF clustering for 524 m^{6}A phenotype-related genes in meta-GEO with cluster numbers from 2 to 6. (B) The relationship between cophenetic, dispersion, residuals, and silhouette coefficients with respect to the number of clusters. (C) Comparison of PD-L1 expression in three m^{6}A gene signature subgroups. (D) The expression of 23 m^{6}A regulators in three gene

signature subtypes. The upper and lower ends of the boxes represented an interquartile range of values. The lines in the boxes represented the median value, and black dots showed outliers. The asterisks represented the statistical P-value (*P < 0.05; **P < 0.01; ***P < 0.001). The one-way ANOVA test was used to test the statistical differences among three gene clusters.

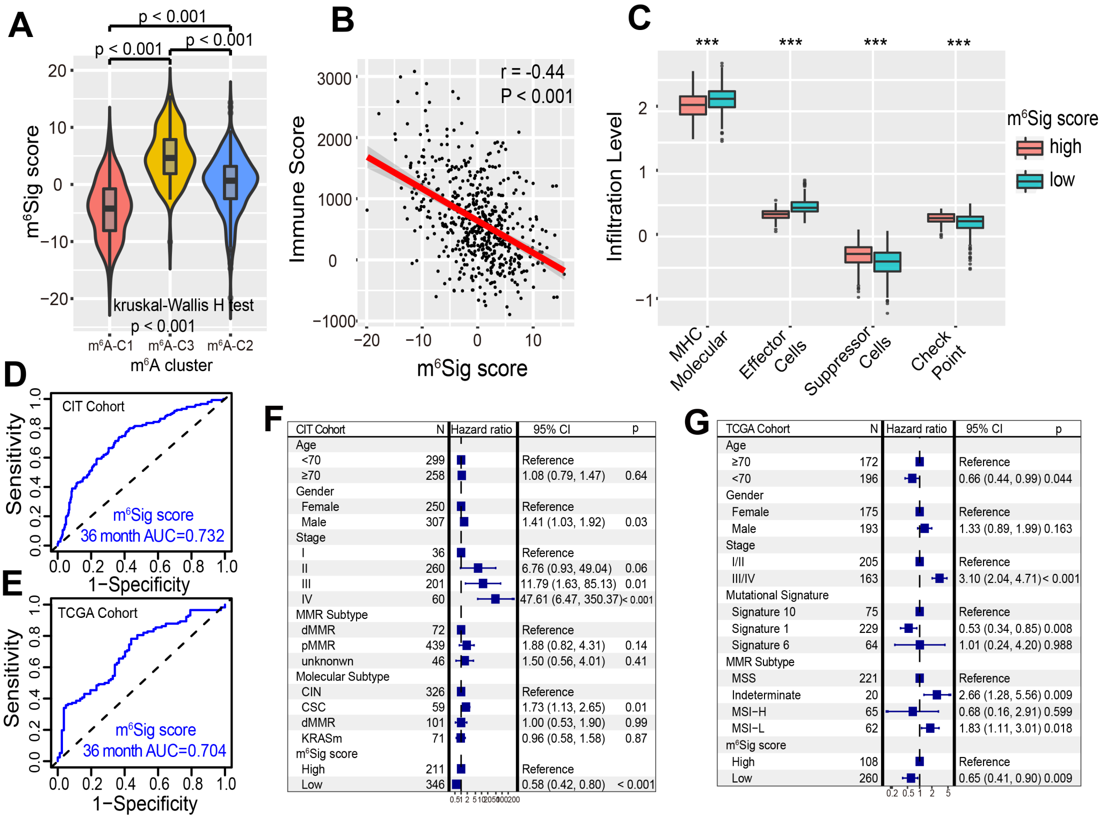

Figure S6. m^{6}Sig score associated with TIM immune regulation and survival outcome. (A) Differences in m^{6}Sig score among three m^{6}A modification patterns (P < 0.001, Kruskal-Wallis test). (B) There was also a significant inverse correlation between the m^{6}Sig score and the immune score (r = -0.44, P < 0.001, Spearman correlation analysis). (C) Compared with patients with a high m^{6}Sig score, the low m^{6}Sig score subgroup exhibited a higher proportion of MHC molecule and effector cells but a lower proportion of suppressor cells and immune checkpoints molecule. (D-E) The predictive value of m^{6}Sig score measured by ROC curves in CIT cohorts (D) and TCGA cohorts (E), AUC = Area under curve. (F-G) Subgroup analysis estimating the clinical prognostic value of m^{6}Sig score in CIT (F) and TCGA (G) cohort by multivariate Cox regression. The length of the horizontal line represented the 95%

confidence interval for each group.

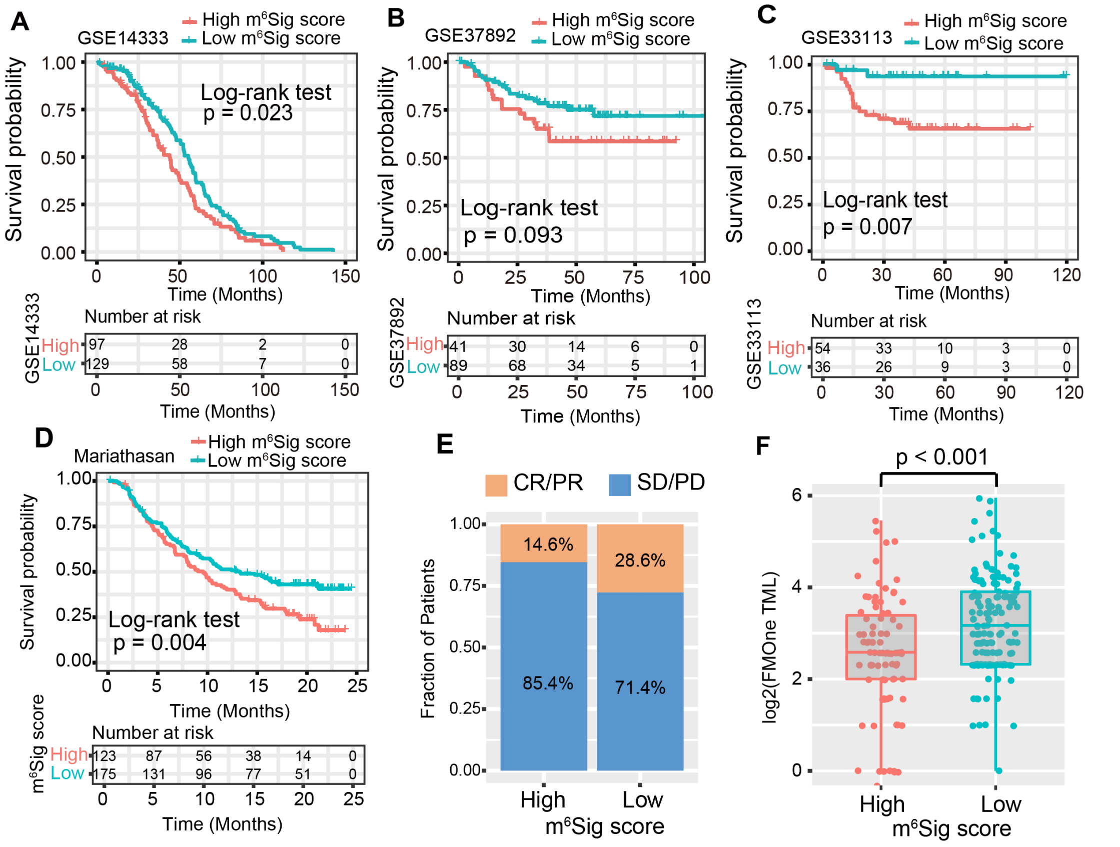

Figure S7. Independent validation of m^{6}Sig score associated with survival outcome. (A-C) Survival analysis of m^{6}Sig score in collected independent colon cancer cohort including GSE14333, GSE37892, and GSE33113 (Statistical significance were calculated by Log-rank test). (D) Kaplan-Meier curves for high and low m^{6}Sig score patient subgroups in the anti-PD-L1 treatment cohort (Mariathasan *et al.* study). Log-rank test, P = 0.004. (E) The fraction of patients with clinical response to anti-PD-1 immunotherapy in low or high m^{6}Sig score groups. CR/PR *vs.* SD/PD: 28.6% *vs.* 71.4% in the low m^{6}Sig score groups, 14.6% *vs.* 85.4% in the high m^{6}Sig score groups. CR, complete response; PR, partial response; SD, stable disease; PD, progressive disease. (F) Relative distribution of tumor mutation load in m^{6}Sig score high versus low subgroups.

Table S1. Summary of clinical characteristics of patients with colon cancer in four datasets.

Table S2. Clinical annotation and m^{6}A modification pattern of the individual

patient in meta-GEO cohorts.

Table S3. Clinical annotation and m^{6}A modification pattern of the individual patient in TCGA-COAD cohort.

Table S4. Identification of significantly mutated genes in colon cancer.

Table S5. Prognostic analysis of 524 m^{6}A-related DEGs using a univariate Cox analysis.
